# Patterns of Arc mRNA expression in the rat brain following dual recall of fear- and reward-based socially acquired information

**DOI:** 10.1038/s41598-023-29609-6

**Published:** 2023-02-10

**Authors:** Laura A. Agee, Emily N. Hilz, Dohyun Jun, Victoria Nemchek, Hongjoo J. Lee, Marie-H. Monfils

**Affiliations:** 1 Department of Psychology, The University of Texas at Austin, Austin, TX USA; 2 Institute for Neuroscience, The University of Texas at Austin, Austin, TX USA

**Keywords:** Fear conditioning, Neuroscience, Amygdala, Prefrontal cortex

## Abstract

Learning can occur via direct experience or through observation of another individual (i.e., social learning). While research focused on understanding the neural mechanisms of direct learning is prevalent, less work has examined the brain circuitry mediating the acquisition and recall of socially acquired information. Here, we aimed to further elucidate the mechanisms underlying recall of socially acquired information by having male and female rats sequentially recall a socially transmitted food preference (STFP) and a fear association via fear conditioning by-proxy (FCbP). Brain tissue was processed for mRNA expression of the immediate early gene (IEG) *Arc*, which expresses in the nucleus following transcription before migrating to the cytoplasm over the next 25 min. Given this timeframe, we could identify whether *Arc* transcription was triggered by STFP recall, FCbP recall, or both. Contrary to past research, we found no differences in any *Arc* expression measures across a number of prefrontal regions and the ventral CA3 of the hippocampus between controls, demonstrators, and observers. We theorize that these results may indicate that relatively little *Arc-*dependent neural restructuring is taking place in the prefrontal cortices and ventral CA3 following recall of recently socially acquired information or directly acquired fear associations in these areas.

## Introduction

An animal’s capacity to survive in a new environment is largely contingent on their ability to learn about and adapt to their surroundings by identifying both potential threats and sources for fulfilling essential needs. One of the primary ways in which humans are able to learn such strategies at an individual level is through instruction by or observation of an experienced individual, i.e., via social learning. As such, it should not be surprising that deficits in the ability to socially learn, such as impairments in the social learning/reward systems observed in autism spectrum disorder, have the potential to significantly impair functioning^1–3^. Conversely, overly indiscriminate social learning can lead to the acquisition of false information or maladaptive behaviors. Clinically, this is often seen in phobias, which are commonly reported to have been acquired through observation or instruction (e.g., watching a parent react with extreme fear to a spider or receiving dire warnings about the danger of spiders, respectively) rather than by direct experience^4,5^. Socially acquired phobias may also be disruptive in ways directly acquired phobias are not. Because the individual has not directly experienced the aversive consequences in relation to the feared stimuli, they are free to imagine an associated outcome that may be more intense than what occurs in reality. In line with this, individuals with socially acquired phobias report increased cognitive symptomology^6^ and respond more favorably to certain treatment methods^4^ than do individuals with directly acquired phobias.

To develop optimal treatments for conditions arising from under- or over-performing social learning, a thorough understanding of the brain mechanisms that underlie the social learning process is an essential first step. In rodents, fear-based social learning has been shown to occur under multiple conditions, including: (1) context or stimulus associated fear acquired by observation through a barrier of a conspecific experiencing pain in a novel environment or following the presentation of a novel stimulus^7,8^, (2) enhanced acquisition of natural behaviors by observation of a conspecific responding to a threatening stimuli^9–11^, and (3) by observation of a fear conditioned demonstrator reacting to the fear-associated conditioned stimulus (CS) post-conditioning in a paradigm known as fear conditioning by-proxy (FCbP)^12–14^. In all cases, observers display fear behavior (e.g., freezing in FCbP) in response to the context or stimulus after observing the demonstrator’s fear response. While similar reward-based models of social learning in rodents have proven somewhat more difficult to develop^15^, one reliable and well-established model of reward-based socially mediated learning does exist in the social transmission of food preference (STFP) paradigm^16–19^. In the STFP paradigm, rats assigned to the ‘demonstrator’ condition consume a novel food (generally powdered chow mixed with flavoring, such as cinnamon) before interacting with a naïve rat assigned to the ‘observer’ condition. When observers are later given the choice to consume either the demonstrated flavor or an entirely novel flavor, they reliably show the tendency to consume more of the demonstrated flavor. This effect has been shown to be mediated by the semiochemical carbon disulfide (CS_2_) which is present in the nasal cavity of rats and, when paired with a novel scent, is sufficient to induce a preference for similarly scented foods^17^.

In rodents, there has been a fair amount of research examining the brain mechanisms mediating the acquisition and recall processes for STFP^20–24^ and socially acquired fears^7,12,13,25–27^. Results from research into the latter topic have also shown that there are a number of brain areas that seem to be uniquely activated during social fear learning and not direct fear learning^13,27^. Integrative models considering the results from both human and non-human animal research into the brain circuitry underlying the social learning of appetitively and aversively motivated behaviors/associations posit that, while there may be considerable overlap between the brain areas governing direct learning processes and social learning processes, activity in some unique brain regions is required for social learning to occur^13,28^.

**Figure 1:**
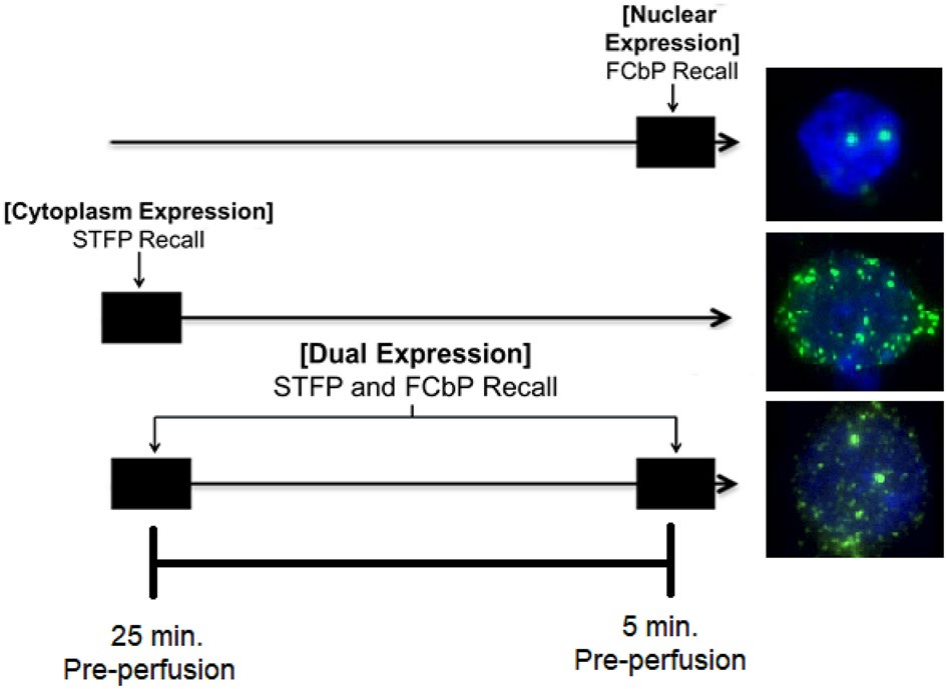
Patterns of *Arc* mRNA Expression. The above figure shows the pattern and area within a cell in which we would see *Arc* mRNA expression triggered by activity at the FCbP recall timepoint, the STFP recall timepoint, or activity that was triggered at both timepoints.

While the neural mechanisms involved in the social acquisition of tasks and information have been explored, research explicitly comparing the storage of memories acquired by social learning to memories acquired by direct learning is, to our knowledge, almost nonexistent. In the experiment described here, we attempted to examine activation in various brain regions following recall of a socially acquired memory from both a reward- and fear-based task. Rats were trained in a reward-based form of social learning, STFP, and a fear-based model of social learning, FCbP, after which we initiated sequential recall of both memories. The tissue from these rats was then processed for mRNA expression of the immediate-early gene (IEG) *Arc* which, when transcribed, produces the mRNA for the activity-regulated cytoskeleton associated (Arc) protein. *Arc* mRNA has a predictable pattern of expression such that in the first 5 min following transcription it is expressed in the nucleus of the cell and, after about 25 min, migrates to the cytoplasm surrounding the nucleus^29^. As such, cells stained for *Arc* mRNA that are activated at both timepoints show expression in both the cytoplasm and nucleus, allowing for precise localization of cell populations activated in multiple tasks (see Fig. 1).

**Figure 2:**
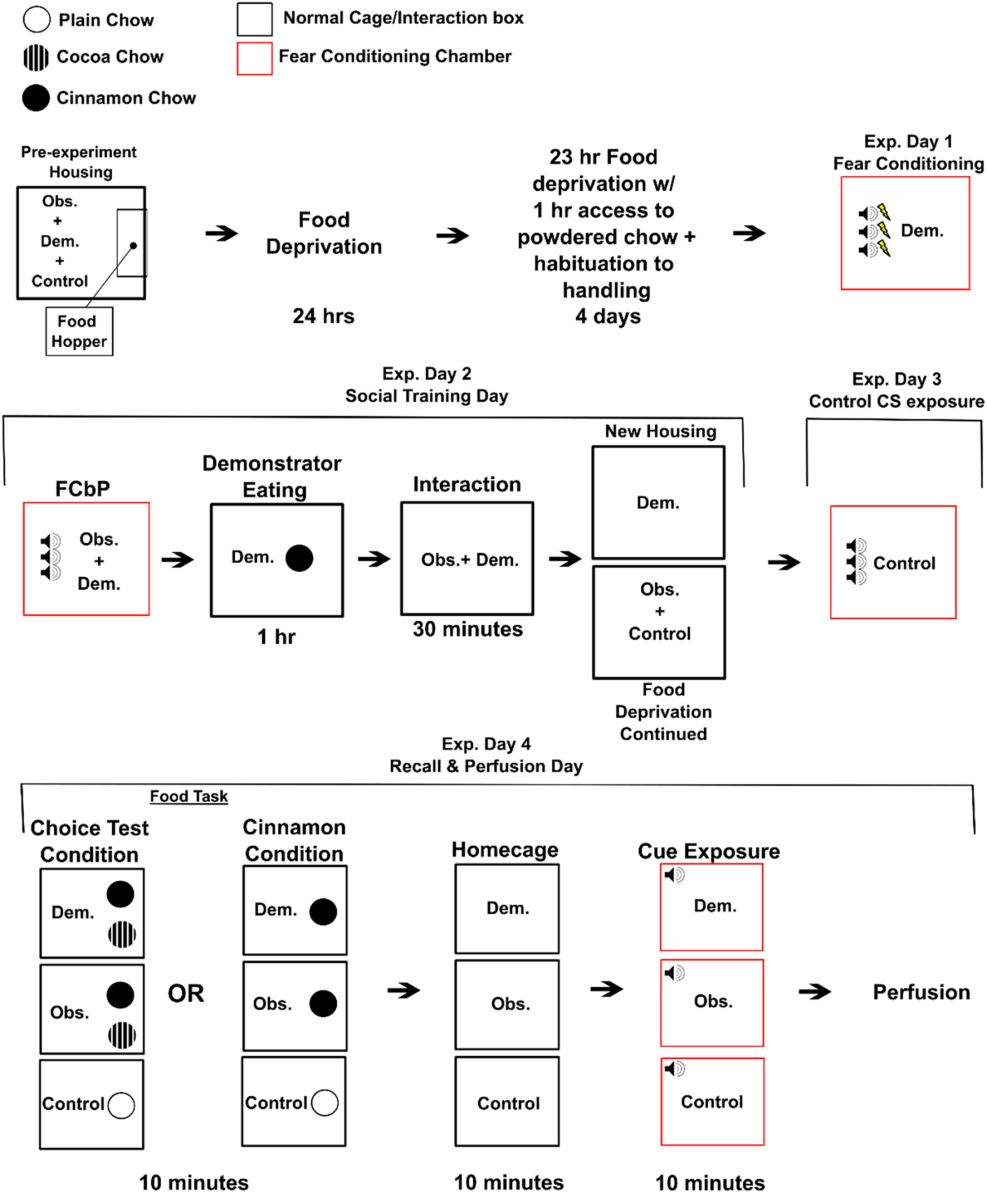
Overview of Experiment Design. This figure outlines the treatment of rats on each day of the experiment from the first day of food restriction on. Following a period of food restriction, demonstrators were fear conditioned to a conditioned stimulus (CS). 24 h later, observers underwent with their demonstrator fear conditioning by-proxy followed shortly by socially transmitted food preference acquisition. Controls received CS exposure the following day with no demonstrator present. Finally, 48 h post-observational learning, all observers and demonstrators underwent a choice test/were given access to the demonstrated food. Controls received plain powdered food for this time period. All rats were then returned to their home cage for 10 min before being returned to the fear conditioning chambers and played a single CS. All rats were subsequently euthanized, and their brains were processed for *Arc* mRNA expression.

By analyzing the expression of *Arc* mRNA in observer rat brains perfused following the sequential recall of FCbP and STFP tasks and comparing them to demonstrators that had gone through recall of analogous direct learning procedures and untrained controls, we aimed to identify brain regions uniquely involved in retrieval of socially acquired information (see Fig. 2 and “Methods” for experimental overview). The anterior cingulate cortex (ACC)^7,13,28^, ventral and lateral orbitofrontal cortices (vOFC and lOFC)^23,24^, the ventral CA3 (vCA3) of the hippocampus^22,23^, and infralimbic and prelimbic cortices (ILC and PLC, respectively)^23,30–32^ were all of particular interest given past research which has implicated them in fear learning, social fear learning, STFP learning, or some combination of the three. We hypothesized that if a region were involved in the storage of socially acquired information specifically, we would see increased levels of *Arc* mRNA in observers as compared to demonstrators and controls. Similarly, we expected demonstrators would show higher levels of *Arc* mRNA in regions specific to storage of directly acquired information. Furthermore, we predicted that both observers and demonstrators would show higher expression compared to controls in regions involved in general memory storage independent of how it was acquired (i.e., socially vs direct. Regions involved most broadly in social and/or individual learning (i.e., regardless of the valence of the information) would show these group differences in the dual expression counts. Finally, the area(s) of expression in which counts differed between groups would tell us whether these differences were task dependent (e.g., only triggered by STFP or FCbP).

## Results

### Behavioral results

#### Fear conditioning and fear conditioning by-proxy

**Figure 3:**
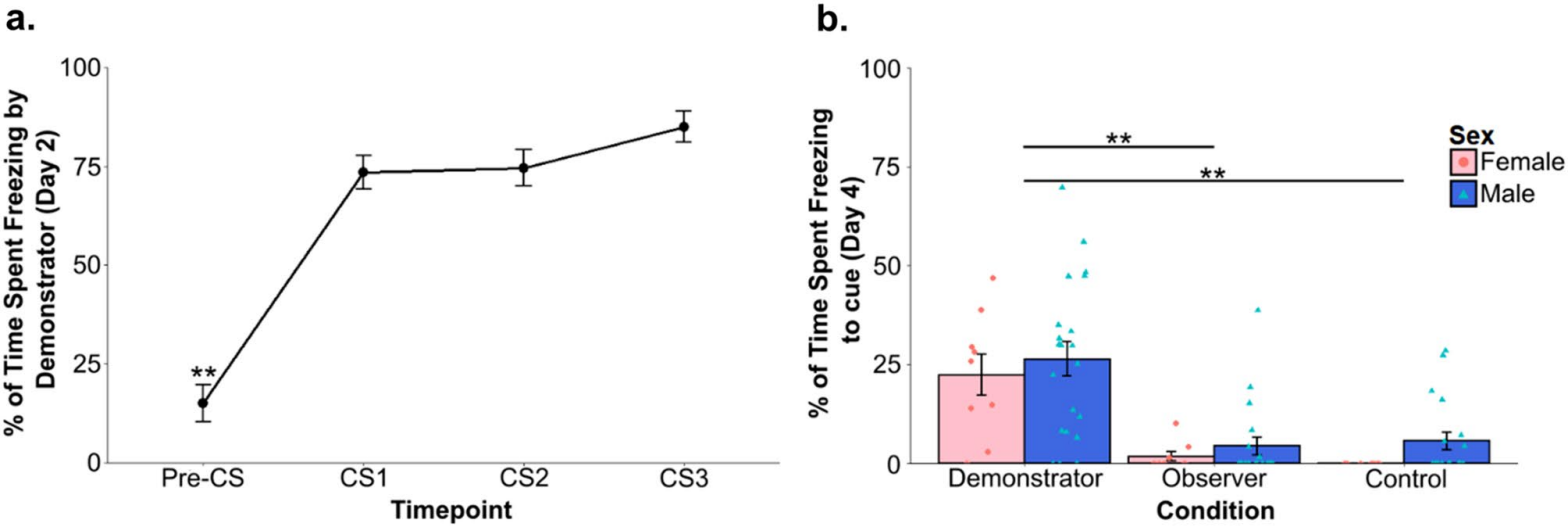
Fear conditioning and fear conditioning by-proxy behavioral results. The above figures show the average percent of total time that rats froze during or prior (Pre-CS) to the CS presentation for (**a**) Demonstrators on day 2, during FCbP interactions and (**b**) all rats to the single CS presentation on the terminal day of the experiment. While demonstrators froze significantly more than both observers and controls, unusually, observers did not freeze significantly more than controls. Values are the mean ± SEM. **p < 0.005.

Demonstrator freezing on day 2 (during the FCbP observation period) was analyzed using one-way within-subjects ANOVA with timepoint (pre-CS, CS1, CS2, and CS3) as the within-subjects factor. We found a significant effect of cue (F_(3,87)_ = 77.96, p < 0.0001) and post-hoc pairwise testing using a Bonferroni adjustment for multiple comparisons confirmed that freezing during the CS was significantly higher than at baseline (all p < 0.001) (Fig. 3a). Kruskal-Wallace analyses were run on freezing on to the CS presentation on the terminal day (day 4) of the experiment as a nonparametric alternative to an ANOVA due to violations of ANOVA assumptions by the untransformed dependent variable. Kruskal-Wallace analyses were run on sex, experimental condition, and a combined sex/condition factor. While there was no overall effect of sex (H_1_ = 1.55, p = 0.2132), there was a significant effect of experimental condition (H_2_ = 35.1, p < 0.0001) and a significant effect of the combined factor (H_5_ = 37.38, p < 0.0001). Post-hoc analyses using Holm’s adjusted Dunn’s tests showed that rats in the Demonstrator condition froze significantly more to the CS than both Observers (p < 0.0001) and Controls (p < 0.0001). Observers and Controls did not significantly differ in their freezing from each other (p = 0.814). Dunn’s testing on the combined sex and condition variable found that the overall effect detected via Kruskal-Wallace was driven entirely by the Demonstrator condition (Fig. 3b). Notably, Demonstrators also displayed an unusually low percentage of freezing to this final CS (mean = 25.2) that we were unable to replicate using near identical behavioral procedures (see Supplementary Materials). We did, however, confirm that the Demonstrators’ freezing during the CS period was not just context-based by using a Wilcoxon signs-rank test (due to violation of the assumption of normality because of a floor effect for pre-CS freezing) to compare freezing during the CS to their freezing prior to CS presentation (Z = 49, p = 0.0013). Surprisingly, and counter to our previous observations^12–14,26^, observers did not show significantly higher freezing to the CS as compared to controls at recall. While concerning, this is likely the result of our using only a single CS presentation. Although past research in our lab has found that FCbP observer rats will freeze over controls on the first CS presentation of a long-term memory test^12^, there were some methodological changes (pre-exposure of controls to the CS and rats being run during their dark cycle) that resulted in slight changes in behavior. This was confirmed in a follow-up experiment run under similar conditions where we ran a full three CS recall test (see Supplementary Materials and Fig. S3).

#### Choice test/food tasks

**Figure 4:**
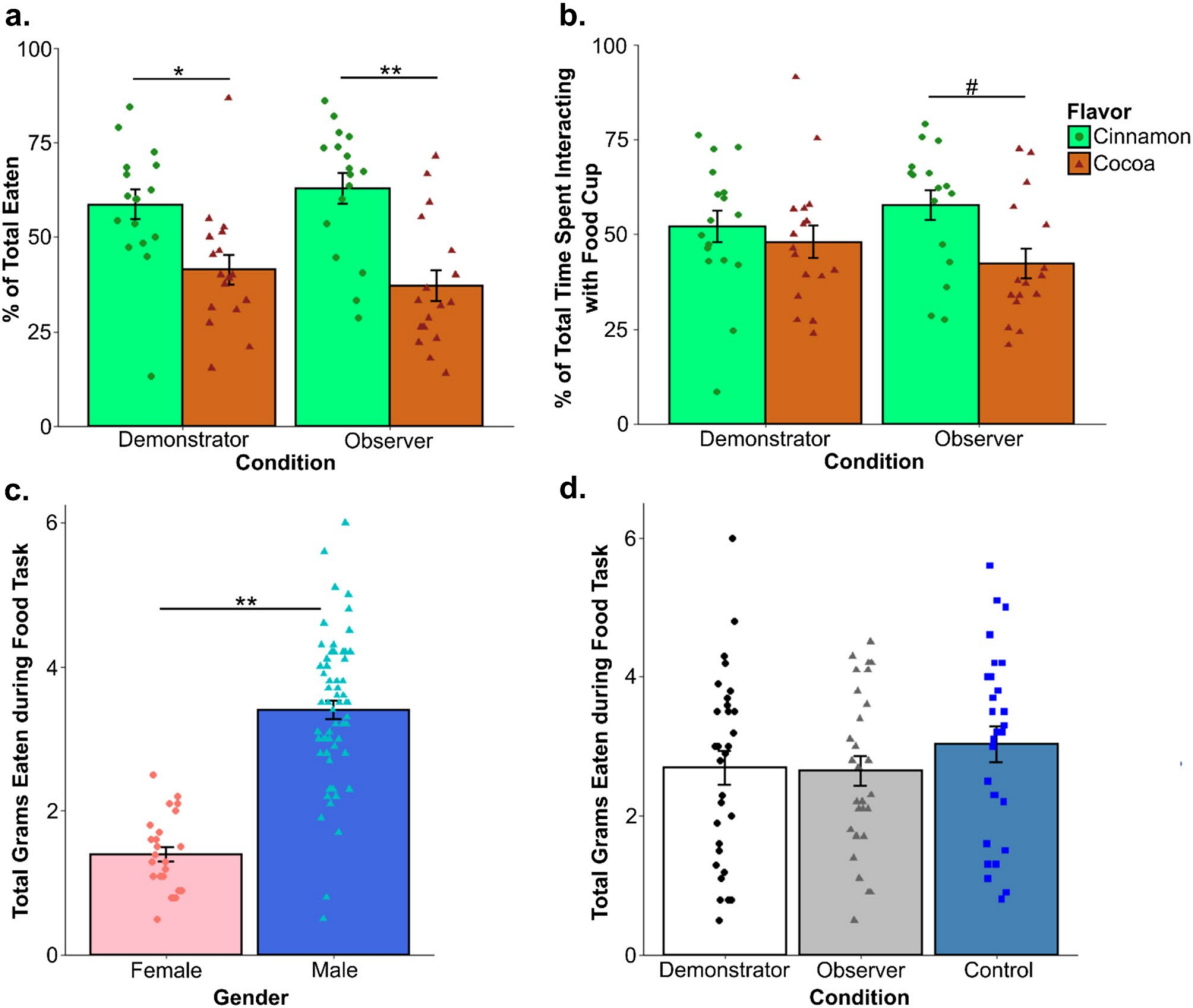
Day 4 food task behavioral results. For rats that went through the choice test on the final day of experimentation, we found that (**a**) while observers and demonstrators did not differ significantly from each other in the percent of diet Cin (the demonstrated flavor) eaten, they did both show a significant preference for the diet. However, (**b**) neither group spent significantly more time interacting with the food cup containing diet Cin. Examining the total amount eaten during the final food task for all rats we predictably found that (**c**) females overall ate significantly less than males. (**d**) Experimental condition has no overall effect on the total amount eaten*.* All graphed values are the mean ± SEM. *#p* < 0.1, *p < 0.05, **p < 0.01.

Choice test performance using either percent of time spent interacting the food cup containing diet Cin or percent of all food eaten that was diet Cin was compared between Demonstrators and Observers using a two-sample t-test. We found no significant difference between the two groups on time spent at the diet Cin food cup (t_31_ = 0.97, p = 0.3404) or on the percent of total eaten that was diet Cin (t_31_ = 0.74, p = 0.4636). To determine whether this lack of an effect was due to both groups showing a preference for diet Cin, we ran a set of one-sample t-tests comparing the percent of total eaten that was diet Cin against the case in which rats showed no preference for either diet (μ = 50). We found that while both Demonstrator (t_16_ = 2.204, p = 0.04265) and Observer (t_16_ = 3.105, p = 0.0068) rats showed a significant preference for the diet Cin based on the percent eaten, neither Demonstrators (t_16_ = 0.476, p = 0.641) nor Observers (t_15_ = 1.885, p = 0.079) spent significantly more time interacting with the diet Cin food cup (Fig. 4a,b). Unfortunately, the non-significance of these findings makes in unclear whether observers actually acquired the preference in this experiment. However, past work from our lab using the same flavored diets and STFP acquisition procedure did produce a robust preference for the demonstrated flavor when the demonstrated flavor was varied and a much longer (18 h) choice test was allowed^33^. The lack of difference between Observers and their Demonstrators can likely be explained by: (1) a slight innate preference for diet Cin over diet Co, as past research in our lab has found in Sprague-Dawleys^14^, (2) our decision to only use diet Cin as the demonstrated flavor in an attempt to decrease variance in the behavioral experience of our observers, and (3) the brevity of the choice test compared to our standard design (10 min vs 1 h). It is also worth noting that the Cohen’s d effect size for the Observer’s preference towards cinnamon (d = 0.75) is larger than the effect size calculated for Demonstrators (d = 0.53), though both fall into the category of medium effect sizes. Finally, we ran a two-way ANOVA with total grams of food eaten during the choice as the dependent variable and experimental condition and sex as the independent variables. We found that while, as expected, there was a significant effect of sex (F_(1,82)_ = 35.66, p < 0.0001) (Fig. 4c) with females eating less than males, there was no significant effect of experimental condition (F_(2,81)_ = 0.334, p = 0.717) (Fig. 4d) and no interaction between the two (F_(2,81)_ = 1.02, p = 0.365).

### Arc results

(An overview of statistical results for each area can be found in S1–S4 Tables. Additionally, see the “Statistical analysis overview” for a detailed description of general statistical procedures).

**Figure 5:**
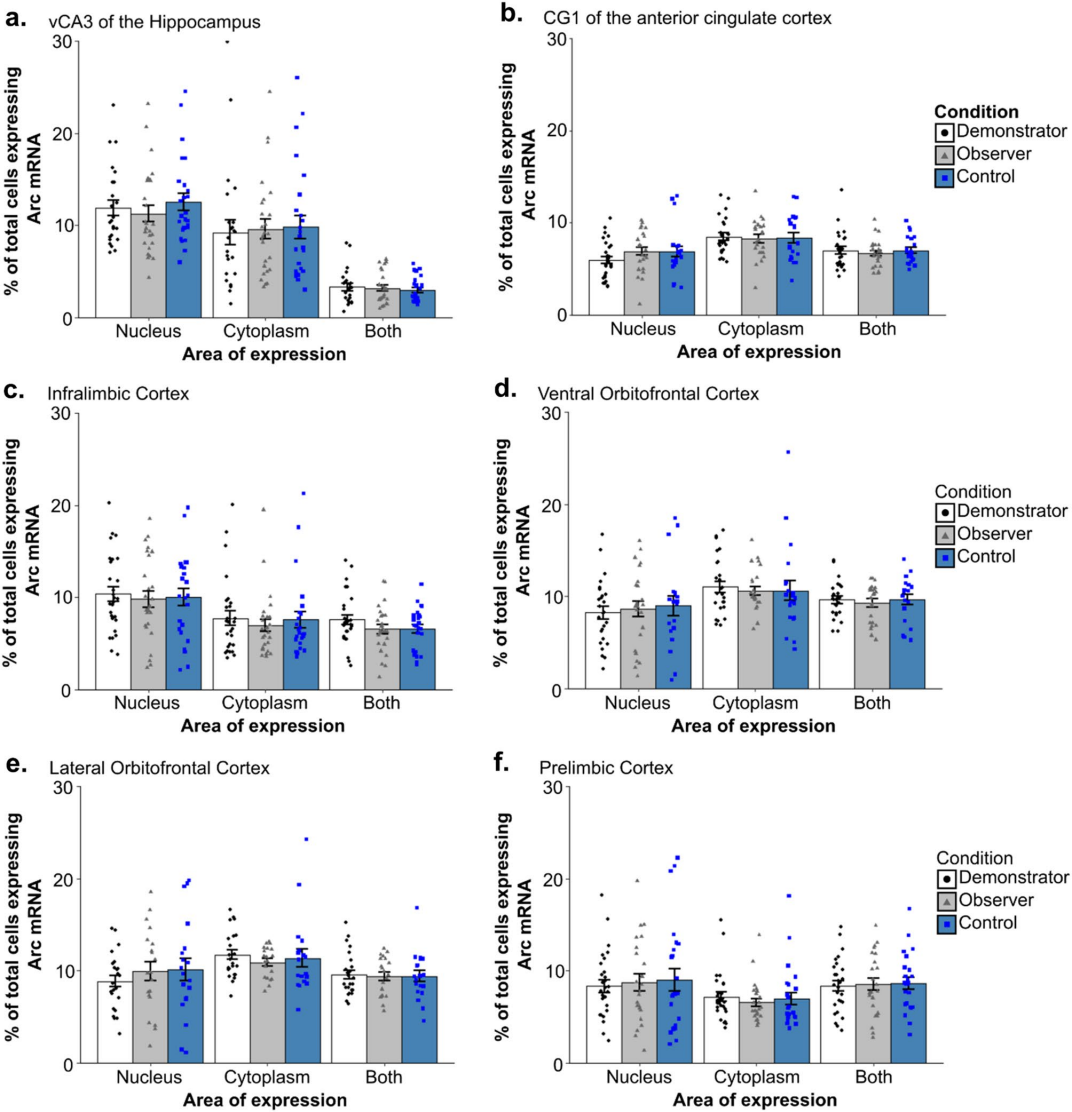
Arc counts across primary experimental condition*.* The above graphs show the percent of total DAPI stained cells that displayed *Arc* expression in the nucleus, cytoplasm, or in both area (dual) across the primary experimental conditions in (**a**) the vCA3 of the hippocampus, (**b**) the CG1 region of the anterior cingulate cortex, (**c**) the infralimbic cortex, (**d**) the ventral orbitofrontal cortex, (**e**) the lateral orbitofrontal cortex, and (**f**) the prelimbic cortex. Across all regions and areas of cell expression examined, no group differences were found between any of the conditions (all p > 0.1). All values represent the mean ± SEM.

**Figure 6:**
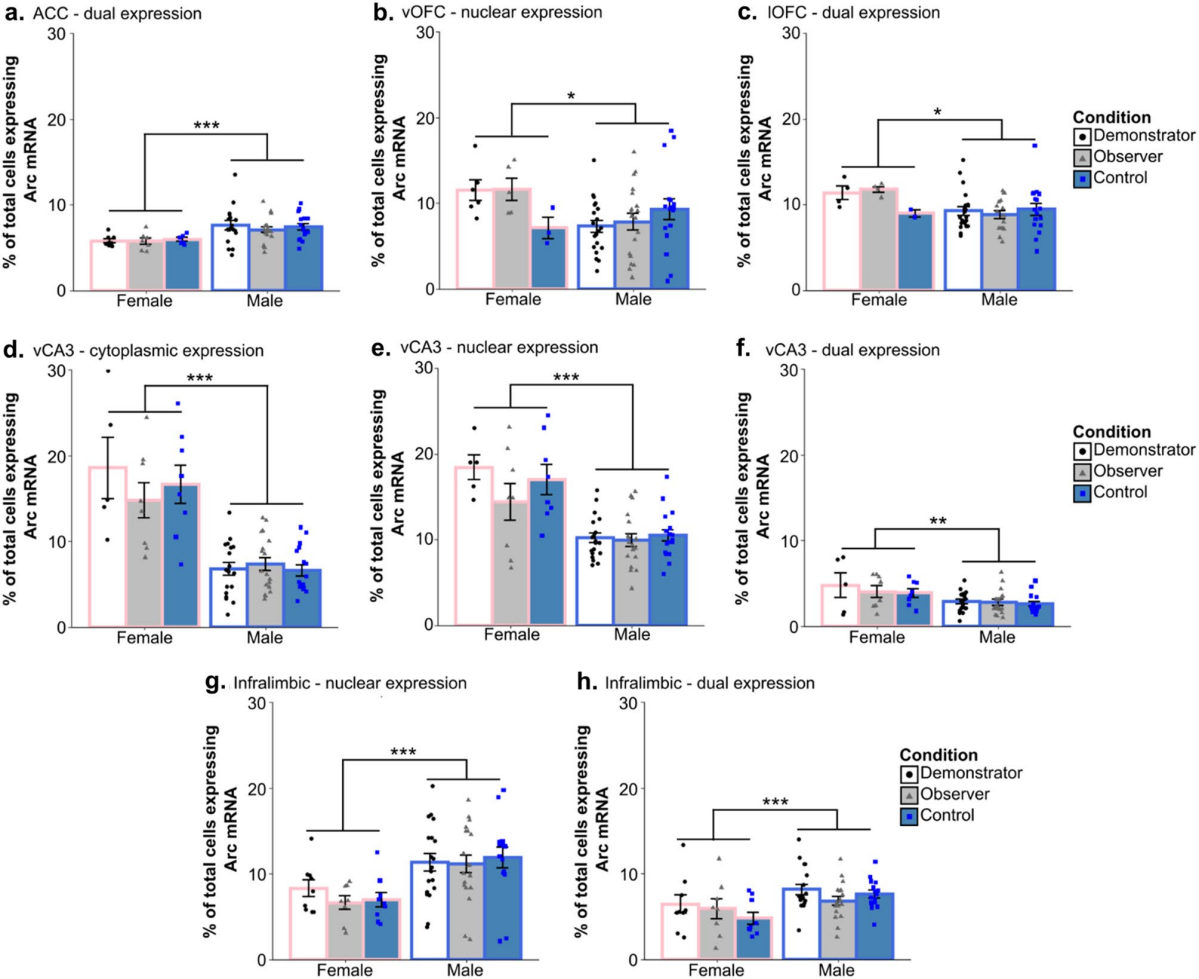
Differences in *Arc* expression between male and female rats. Significant differences in *Arc* expression were seen between male and female subjects when comparing (**a**) dual *Arc* expression in the CG1 region of the anterior cingulate cortex, (**b**) nuclear *Arc* expression in the ventral orbitofrontal cortex, (**c**) dual expression in the lateral orbitofrontal cortex, (**d**) cytoplasmic, (**e**) nuclear, and (**f**) dual *Arc* expression in the vCA3 of the hippocampus, and (**g**) nuclear and (**h**) dual *Arc* expression in the infralimbic cortex. Values represent the mean ± SEM. + *p* < 0.1, *p < 0.05, **p < 0.01, ***p < 0.001.

**Figure 7:**
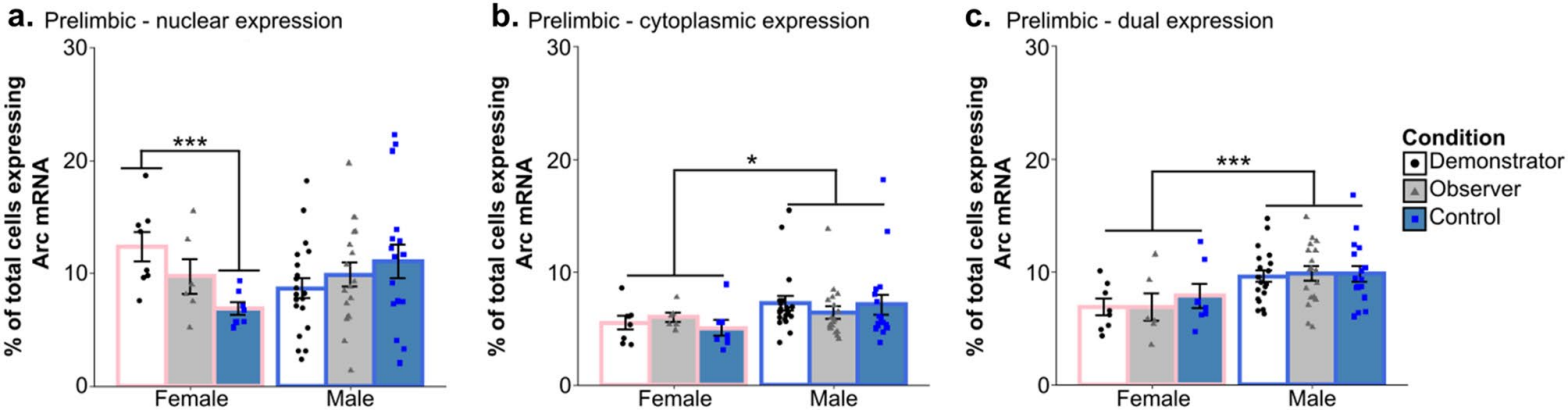
Differences in *Arc* expression between male and female rats across the prelimbic cortex. Initial ANOVA analysis found a significant sex and condition interaction in (**a**) the nuclear prelimbic counts, with female Demonstrators showing significantly more *Arc* expression than female Controls. Compared to males, females had lower overall (**b**) cytoplasmic and (**c**) dual *Arc* expression in the prelimbic cortex. Values are the mean ± SEM. *p < 0.05, ***p < 0.001.

**Figure 8:**
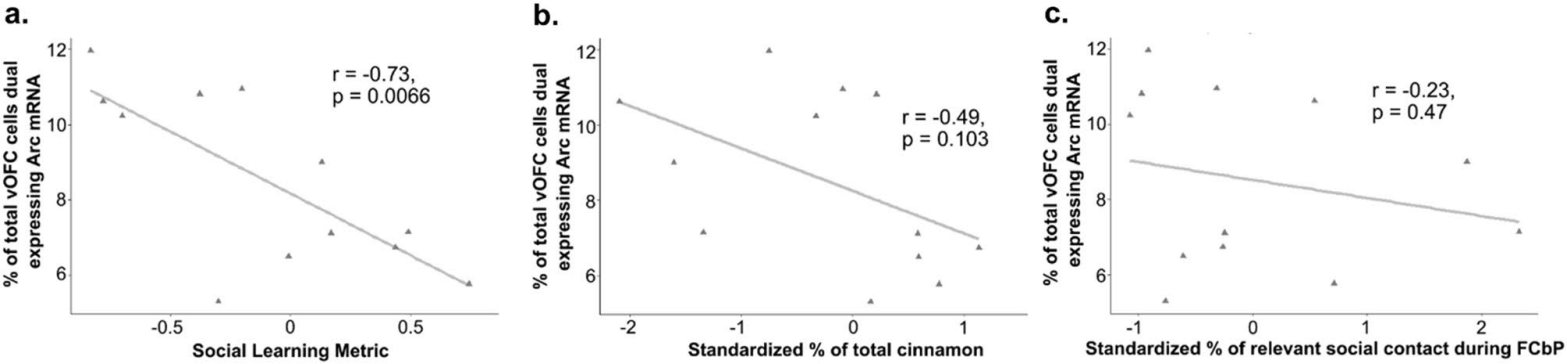
Relationship between social learning measures dual *Arc* expression in the vOFC. (**a**) A significant negative relationship was found between a social learning metric calculated by summing standardized measures of social acquisition of the STFP and socially acquired fear association in Observers and the percent of *Arc* dual-expressing cells in the ventral orbitofrontal cortex. This relationship was not significant when looking at either (**b**) the standardized measure of STFP or (**c**) the standardized measure of sex relevant social contact during FCbP—used as a proxy for social fear learning—alone. Notably, both male and female rats were included in this dataset.

Two-way ANOVAs found no effect of condition and no interaction between sex and condition was detected in the vCA3, ILC, ACC, lOFC, or vOFC (all p > 0.05) (Fig. 5). Additionally, none of the one-way ANOVAs detected effects of condition when rats were further separated based on the food task they were assigned in any of these areas or in the PLC (all p > 0.1). An overall effect of sex was found in a number of regions including: nuclear *Arc* expression in the vOFC (F_(1,64)_ = 4.851, p = 0.031; η^2^_partial_ = 0.07); dual expressing cells in the lOFC (F_(1,57)_ = 6.18, p = 0.016, η^2^_partial_ = 0.094); nuclear (F_(1,69)_ = 35.470, p < 0.001, η^2^_partial_ = 0.325), cytoplasmic (F_(1,69)_ = 60.715, p < 0.0001, η^2^_partial_ = 0.463), and dual expressing (F_(1,69)_ = 9.84, p = 0.003, η^2^_partial_ = 0.124) cells in the vCA3 of the hippocampus; dual expressing cells in the CG1 region of the ACC (F_(1,66)_ = 15.930, p < 0.001, η^2^_partial_ = 0.194); in nuclear expressing cells (F_(1,73)_ = 18.05, p < 0.001, η^2^_partial_ = 0.196) and dual expressing cells (F_(1,73)_ = 13.666, p < 0.001, η^2^_partial_ = 0.15) in the ILC (Fig. 6); and in cytoplasmic expressing cells (H_1_ = 4.3, p = 0.038, η^2^_H_ = 0.045) and dual expressing cells (F_(1,70)_ = 18.11, p < 0.001, η^2^_partial_ = 0.203) in the PLC (Fig. 7b,c). Female rats displayed higher *Arc* counts than males in areas other than the ACC, ILC, and PLC, in which male counts were higher across all conditions. Notably, post-hoc analyses found no overall significant effect of condition within the *Arc* counts across any of the tested regions or areas of cell expression (all p > 0.1). The two-way ANOVA examining nuclear expression in the PLC found a significant interaction effect between sex and experimental condition (F_(2,70)_ = 3.96, p = 0.023, η^2^_partial_ = 0.102). Post-hoc testing found a significant difference between nuclear *Arc* expression in female Demonstrators and female Controls only (t_9.7_ = 3.9, p = 0.0032, d = 1.22) (Fig. 7a). Correlational analyses found a significant negative relationship between the social learning metric (SLM) score—a combined measure of estimated social learning success (see “Statistical analysis overview” in “Methods”)—of Observer rats and the percent of cells showing dual *Arc* expression in the ventral orbitofrontal cortex (t_10_ = − 3.41, p = 0.0066, r = − 0.73) (Fig. 8a). Follow up analyses confirmed that this relationship was not significant when looking at either the standardized measure of percent cinnamon eaten (t_10_ = − 1.795, p = 0.103, r = − 0.49) or the standardized measure of sex relevant contact during FCbP (t_10_ = − 0.75, p = 0.47, r = − 0.23) alone (Fig. 8b,c). All other correlational analyses were not significant beyond our Bonferroni corrected alpha value (all p > 0.01).

## Discussion

Contrary to our expectations, our results did not show any differences in *Arc* expression following long term memory recall based on whether the subject had acquired reward- and fear-based information by means of direct learning or social learning. Control rats that underwent analogous behavioral procedures prior to euthanasia but that had not received any explicit fear- or reward-based training did not differ in *Arc* expression across the CG1 region of the ACC, the ILC, the vCA3 of the hippocampus, the vOFC, or the lOFC when compared to Demonstrators or Observers. With the exception of the PLC in females, the only differences in *Arc* expression that were detected were driven by subjects’ sex and showed no interaction with experimental condition. Though it is true that recall may not necessarily induce as many of the long-term changes in neural activity and connectivity that *Arc* is thought to be involved in^34^ as learning does, past research has found certain recall procedures to be sufficient to induce increased *Arc* activity^35,36^. As such, the lack of an effect across conditions that we see cannot be attributed only to using the recall timepoint. Here, we will first examine our overall findings in the context of past research into the brain mechanisms underlying recall in the STFP paradigm, fear-conditioning and observational fear-conditioning procedures, and our findings in the vOFC in the context of past research. We will then cover our findings—and the limitations around our ability to interpret these findings—on sex differences in *Arc* expression.

### *Arc* in the recall of a socially transmitted food preference

Past research examining expression of the IEG c-Fos has found that a number of the areas we examined, specifically the OFC, vCA3, ILC, and the PLC^22,23^ show activation at the 48 h recall timepoint for a STFP. It is notable that these results from Smith et al.^23^ were obtained using the same STFP control paradigm as was used in this study, indicating that though STFP recall induced activity in these regions may have been detectable with c-Fos, this may not be the case at this timepoint when examining *Arc*. This interpretation is backed up by the findings of Pilarzyk et al.^36^, who examined *Arc* mRNA activity following STFP recall in Pde11a knockout mice, which display impaired recent STFP and enhanced remote STFP compared to wild-type controls. They found that both strains showed increases in *Arc* expression over home-cage controls at this timepoint in the ventral and dorsal CA1, the ventral and dorsal subiculum, and in the CG1 and CG2 of the ACC. Moreover, while Pde11a knockout mice showed decreased *Arc* expression following a recall procedure for a recently acquired (24 h post) STFP memory when compared to wild-types in the vCA1, no difference between the two genetic lines was evident in any of the other regions examined. At a more remote recall timepoint (7 days post), knockout mice showed higher *Arc* activity post-recall in the CG1 and CG2 of the ACC but not in the vCA1 as compared to the wildtype controls. Home cage mice showed no baseline difference regardless of genetic line. These findings are particularly interesting given that past studies have found no differences in c-Fos activity in these areas when recall was induced on the exact same timeframe^22,23^. With this in mind, it is perhaps unsurprising that we also observed no recall induced changes in *Arc* expression in the regions we examined despite their consistently being shown to be active using c-Fos as an activity marker. Exactly what the implications of this are—outside of the obvious conclusion that not all IEGs are equal—is hard to say when working with mostly null findings. That said, the high sensitivity of cellular compartment analysis of temporal activity by fluorescence in situ hybridization (catFISH) and our large group sizes for the primary behavioral conditions does lend validity to the non-significance of our findings. It is also worth noting that we were not able to definitively show STFP acquisition in our observer rats. This is likely due to (1) the abbreviated nature of the choice test and (2) the lack of an entirely naïve control group to which to compare our observers. If acquisition of the STFP was in fact unsuccessful, this could explain our null findings. However, in our previous research, the same acquisition procedure did produce significant increases in preference for the demonstrated flavor as detected by longer choice test procedures^14,33^. As such, it is most likely that acquisition and recall were successful in the present experiment, but the lack of ideal behavioral controls and the short choice test procedure meant that we were unable to definitively show preference for the demonstrated flavor.

### *Arc* in the recall of direct and socially acquired fear associations

Our ability to interpret our findings regarding our rats undergoing recall of fear acquired via direct learning is significantly aided by how well-characterized the system underlying fear learning and recall is. A number of the areas we examined are involved in fear or extinction learning (the latter of which we would assume to be initiated in Demonstrators, as they had undergone non-reinforced CS presentation during FCbP) specifically the ACC, PLC, and ILC^30–32,37^. Though a slightly smaller pool of research is available regarding the neural mechanisms of social fear, the proposed models of social fear learning posit that similar systems underlie social and direct fear learning^28^. Our findings indicate no overall role of the ACC, PLC, or ILC in either recall of a socially acquired fear association or a directly acquired fear association (though see also “Discussion” of sex differences in PLC activity below). However, as covered in the previous section, this likely just indicates that *Arc* does not serve as a reliable indicator of activity in this case. Examination of these areas post-fear acquisition would likely tell a different story. Though explicit research in *Arc* activity following fear recall is limited, there is some past research to draw from. Chia and Otto^35^ found that in trace fear conditioning rats had significantly higher Arc protein expression in both the dorsal and ventral hippocampus when compared to unconditioned controls. Notably, Arc was quantified by Western Blot analysis of the homogenized ventral and dorsal hippocampus in this experiment, so precise localization of hippocampal activity was not available. These findings likely indicate that, as in STFP, *Arc* transcription might be induced in certain areas of the hippocampus at the 48-h recall timepoint for a cued fear memory. Our null results may also potentially be due to failed recall on the part of observers, as unlike in previous studies in our lab^12–14,26^ we did not see higher freezing to the CS in observers as compared to controls. Whether this is actually due to failed recall in observers or due our single CS recall procedure and the pre-exposure of controls to the CS is not clear.

### Potential role of the ventral orbitofrontal cortex in recall of socially acquired information

In a landmark study, Lesburguères et al.^24^. were able to demonstrate that while dorsal hippocampal (dHPC) activity was necessary for acquisition and short-term recall of an acquired STFP, the STFP memory was eventually offloaded to the OFC for long-term storage. Additionally, Lesburguères et al. were able to demonstrate that that tagging of neurons in the orbitofrontal cortex during STFP acquisition is necessary for long-term storage of socially transmitted food preferences and that interference with the OFC activity following acquisition impairs memory recall 30 days post-acquisition (though see also^38^). These findings would suggest communication between the dHPC and the OFC in the first days or weeks following STFP acquisition and ongoing reorganization of the OFC at this timepoint to accommodate the long-term storage of the STFP memory. While the lack of overall differences in ventral or lateral OFC *Arc* expression between Demonstrators, Controls, and Observers in this study would challenge that interpretation, we did detect a significant negative correlation between our combined measure of overall social learning performance and dual-*Arc* expressing cells in the vOFC. Furthermore, this correlation was not observed between a similar metric formed for Demonstrators based on choice test performance and freezing to the CS. As reliance on socially acquired information can be thought of as making the choice between potentially unreliable social information and the dangers of learning through direct experience, it is possible that this apparent inhibitory role of the vOFC on expression of socially acquired information might be connected to the OFC’s broader role in value-based decision making^39–42^.

### Sex differences in ***Arc*** transcription

Prior to this discussion, it should be stated that our ability to interpret our sex-related results is hindered for a number of statistical and methodological reasons. First, our occasionally low sample size for females, with group size for sex/condition combinations ranging from n = 2 to n = 9 following removal of rats without enough viable sections (though notably an n < 5 was only present for female Controls in the vOFC and lOFC and female Demonstrators and Observers in the lOFC, see Table S5 for specific group sizes). Additionally, our lack of entirely undisturbed controls means that we have no way to determine whether these sex differences are the result of baseline or task-specific differences in *Arc* mRNA production. Finally, because the pre-in situ PFA wash was not introduced until all female sections had been processed, it is possible that this difference in tissues processing might have affected the overall stain. That said, if this were the case, we might expect to see a broader and more consistent effect of sex across regions and types of *Arc* expression (nuclear, cytoplasmic, and dual). As it is, 18 regions/cellular areas of *Arc* expression combinations are examined and only 10 display a significant overall effect of sex. Furthermore, this effect is not uniform in its direction, with males displaying greater overall *Arc* expression in 5 cases and females displaying greater expression in the other 5. Regardless, we feel that our findings here should serve only to inform possible future research into sex differences in *Arc* expression. As it is, the limitation of the current study would make drawing definitive conclusions regarding sex effects on *Arc* expression inappropriate. This should be kept in mind in reading the following discussion.

Although there has been little investigation into sex differences in *Arc* expression, there are some findings indicating that female rats express more *Arc* in certain regions of the dorsal hippocampus following repeated exposure to a relatively enriched environment^43^, though a trend in the opposite direction has also been observed in rats tested without prior behavioral intervention^43^. Our findings may indicate that sex differences in *Arc* transcription are present following certain general behavioral tasks or experiences. In the CG1 region of the ACC we found that males, overall, had more cells active at both timepoints, possibly due to higher baseline *Arc* transcription in the ACC of males or increased transcription following context changes/re-exposure (home cage → STFP testing room → conditioning chamber) as there is some evidence—though limited—for a role of the ACC in long-term recall of contextual memories^44^. Male rats also displayed higher nuclear and dual *Arc* counts in the ILC. This might be explained by the role of the ILC in extinction and fear inhibition^31,45,46^ and observed impairments in the inhibition and extinction of learned fear in females^47–49^. If this is the case, it does raise the question of why no overall differences were observed between our Control, Observer, and Demonstrator rats if *Arc* expression was being triggered by CS-elicited infralimbic activity.

Females showed higher levels of *Arc* expression for all counts in the vCA3. The difference in nuclear counts could potentially have been the result of greater activation following exposure to the CS or re-exposure to the conditioning chamber in females, while the higher levels of cytoplasmic *Arc* expression in the vCA3 following the food task may indicate a sex differences in the role of *Arc* in the vCA3 either the recognition of “familiar” food (even for Observers the scent would be familiar due to their prior interaction with the Demonstrator) or reward/general consummatory processes. That females also showed significantly higher dual labelling in the vCA3—though this effect was small—might also indicate generalized increases in vCA3 *Arc* transcription in females. Female rats also displayed higher nuclear *Arc* transcription in the vOFC and higher dual levels of *Arc* mRNA in the lOFC, though these results are more difficult to interpret due to the low number of female Control rats whose brain tissue was intact enough to take OFC counts (n = 2 and 3 for the lateral and ventral OFC, respectively). Data from the available Control rats indicates a possible sex mediated increase in OFC *Arc* mRNA production*,* but it is just as possible that this effect would not persist with a higher n. It is notable that some past research has indicated structural differences in the OFC and functional differences in OFC-mediated behaviors between female and male rodents^50–52^.

Possibly our most interesting sex differences in *Arc* mRNA were detected in the PLC. There, males showed overall higher numbers of cells expressing *Arc* in the cytoplasm and in both the cytoplasm and nucleus (dual expressing) than females. We also found that while male Demonstrators and Observers did not show increases in *Arc* transcription over Controls at the fear-recall timepoint, female Demonstrators showed significantly higher nuclear *Arc* transcription than Controls while female Observers fell in the middle between the two. This sex-effect may be driven by the aforementioned deficits observed in learned fear inhibition and extinction that are observed in females^47–49^, as past research has suggested that the PLC is critically involved in stimulating fear behavior^45,53,54^. Furthermore, a number of studies have implicated differences in PLC signaling and structure as potential driving factors for these sex-specific impairments in fear-inhibition and extinction^55–58^. While we found no significant difference in female and male freezing behavior to the cue, the upregulation of *Arc* mRNA in response to a non-reinforced fear associated CS in specifically female Demonstrators may be indicative of differential neural restructuring in the PLC that could ultimately lead to sex differences in fear expression.

## Conclusions

While the findings of this study did not broaden our understanding of the brain mechanisms involved in the retrieval of socially acquired memories as much as we had hoped, our results do provide some potential insights on sex differences in *Arc* expression as well as the role (or lack thereof) of *Arc* in long-term memory recall. Our findings suggest that—at least in the prefrontal cortex and vCA3—the induction of brain activity through recall of socially acquired information does not appear to be sufficient to cause increases in *Arc* expression over those caused by the testing procedure alone. However, the validity of this takeaway is certainly brought into question by the inconclusive results of our behavioral tests, as poor retainment of the socially acquired information could be at fault for this lack of effect. We theorize that this might be because minimal neural restructuring is triggered when recall occurs prior to systems consolidation. Further research into the role of the Arc protein in social learning recall processes is still warranted given that our behavioral results do not definitively demonstrate social learning in Observer rats. Future research examining overlap in the neural mechanisms governing different forms of social learning might also benefit from the inclusion of animals undergoing acquisition procedures and animals undergoing remote recall procedures, as these timepoints may be more likely to induce plasticity changes and thus changes in *Arc* expression.

## Methods

### Subjects

Subjects were male and female Sprague–Dawley rats bred in house in the Animal Resource Center of the University of Texas at Austin. Seven breeding pairs produced the subjects for Cohort 1 (n = 27 females, n = 36 males), eight breeding pairs produced the subjects for Cohort 2 (n = 27 males, no females). Female breeders were Sprague–Dawley rats (between 215 and 260 g at arrival) obtained from Charles-Rivers (Wilmington, MA, USA) while male breeders were Sprague–Dawley rats (between 230 and 300 g at arrival) obtained from Harlan (now Envigo) (Houston, TX, USA). All rats were housed with an opposite-sex cage mate until the female showed clear signs of pregnancy, at which point females were singly housed. Pups were weaned into triads of same-sex siblings at post-natal day 21 (P21) to help ensure social fear learning^26^. Spare pups were weaned into triads or dyads with unrelated rats and used in other experiments at the University of Texas at Austin. Female pups from our second cohort litter were used in other experiments. Pups were allowed to mature with minimal disturbances aside from routine animal husbandry procedures (e.g., cage changes) until habituation procedures (Females triads) or dominance assessment procedures (Male triads) began (dominance procedures and results detailed in Supplementary Materials). Cohort 1 rats began procedures between P106–P112 days of age and Cohort 2 rats started between P99–P118 days of age. All subjects were kept on a 12-h reverse dark–light cycle with lights off at 3 PM. All experimental procedures were completed during the subjects’ dark cycle under red light. All parts of this experiment were conducted in compliance with the National Institutes of Health Guide for the Care and Use of Experimental Animals and were approved for use by The University of Texas at Austin Animal Care and Use Committee.

### Apparatus and stimuli

#### Fear conditioning

All fear conditioning and fear conditioning by-proxy procedures were completed in standard conditioning chambers (30.48 cm × 25.4 cm × 30.48 cm) constructed of clear plexiglass walls in the front and back, two steel walls on the side, and a plexiglass ceiling with a hole in the center. The flooring of the chamber was a row of stainless-steel rods connected to a shock generator (Coulbourn Instruments, Allentown, PA). All chambers were enclosed in acoustic isolation boxes (Coulbourn Instruments) and lit with an internal red light. Behavior was recorded by closed-circuit cameras (Panasonic™ WV-BP334) mounted above the conditioning chambers with the lens inserted through the hole in the plexiglass ceiling. Chambers were fully wiped down with 70% alcohol solution between each subject. All stimulus delivery was controlled using the Freeze Frame software (Coulbourn Instruments). The CS was a 20 s tone (5 kHz, 80 dB) and, in procedures with multiple CS presentations, a variable inter-trial interval (ITI) averaging 180 s. The unconditioned stimulus (US) was a 1 mA shock that was 500 ms in duration and co-terminated with the conditioned stimulus.

#### Social transmission of food preference

All STFP procedures took place in a room adjacent to the room containing the conditioning chambers. Novel diets were composed by mixing 100 g of powdered 5LL2 Purina rodent chow with either 1 g of McCormick ground cinnamon (diet Cin) or 2 g of Hershey cocoa powder (diet Co). The Plain diet, which was given to all rats during the food restriction period and to Control rats on the terminal day of experimental procedures, was unadulterated powdered 5LL2 Purina rodent chow. All powdered chows—both during food restriction and experimental procedures—were presented in hanging food cups that were constructed from 4 oz. glass jars and 12-gauge steel utility wire. Food cups were rinsed then wiped down with a 70% ethanol solution before being washed thoroughly with soap and water between every use. All consummatory phases of the STFP experimental procedures took place in standard rat cages (26.7 cm × 48.3 cm × 20.3 cm), with every rat receiving a fresh cage. The interaction phase (STFP acquisition phase) took place in a large plastic bin (50.5 cm × 39.4 cm × 37.5 cm) with wood chip floor bedding that was replaced between every group. Plastic bins were wiped down thoroughly with Windex between each session.

### Overview of experimental design and social learning procedures

(See Fig. 2 for a graphical overview).

All rats were food restricted for five days and habituated to handling and the room where STFP procedures would take place for four days immediately prior to day 1 of experimental procedures. While habituation procedures ended prior to day 1 of experimental procedures, food restriction continued through to the end of the experiment. One rat from each triad was assigned to one of three conditions: Demonstrator, Observer, or Control. Cohort 2 male triads had been assessed for dominance and all showed a clear hierarchy and were assigned such that the dominant rat was the Demonstrator and a subordinate was the Observer to enhance social transmission of fear^13^. Individual triads were further randomly subdivided into groups where the Demonstrator and Observer would receive a choice test at STFP recall (Choice) and groups where they would receive only the demonstrated food (Cin).

On day 1 of the experimental procedure, rats assigned to the Demonstrator condition were moved to fear conditioning chambers and allowed to habituate for 10 min before they were exposed to 3 CSs that co-terminated with a painful shock (see “Apparatus and stimuli” for specifics). Following fear conditioning procedures, Demonstrators were moved back to their original home cage. On day 2 of experimental procedures, 24 h after fear conditioning, Demonstrators were returned to the conditioning chambers with their cage-mate assigned to the Observer condition and put through the FCbP procedure (Fig. 2). Immediately following the FCbP procedure, Observer rats were returned to their home-cage while Demonstrators were moved to an adjacent room and given 1 h to consume powdered chow flavored with cinnamon. After an hour had passed, Observers were moved to an interaction bin with their Demonstrator and allowed to interact with them for 30 min to allow for acquisition of a socially transmitted food preference. Previous research from our lab has validated these timepoints as being sufficient for STFP transmission^33^. Afterwards, Observer rats were returned to their home-cage while Demonstrator rats were moved to single housing to prevent further STFP transmission to the Observer or Control. On day 3 of experimental procedures, Control rats were moved to conditioning chambers alone and, following 10 min of habituation to the chamber, were presented with three 20 s CSs with no accompanying shock. This was done on a separate day to minimize the possibility of lingering alarm pheromones—which are known to be released by rats in response to threatening stimuli and effect conspecific learning^59^—still being present in the chamber.

On the terminal day of the experiment, day 4, recall was initiated for both the socially transmitted food preference and the fear conditioning/fear conditioning by-proxy memories. All Observers and Demonstrators from triads assigned to the Choice condition were allowed 10 min ad libitum access to both cinnamon and cocoa flavored diets, while Observers and Demonstrators from triads assigned to the Cin condition were given 10 min ad libitum access to the cinnamon diet only. In all triads, Control rats were given 10 min ad libitum access to plain powdered chow. Immediately after this, rats were returned to their home-cage and left undisturbed for a 10-min period before being moved back to the lab space and being placed in the conditioning chambers. All rats were then given a 3-min habituation period to the chamber before being presented with a single 20 s CS. 5 min after the end of the CS, all rats were euthanized via injection of a pentobarbital and phenytoin solution (Euthasol; Virbac Animal Health) and perfused. Their brains were later processed for *Arc* mRNA expression. Given the time course of our terminal procedure and the known migration timeframe of *Arc* mRNA^28^, increases cytoplasmic expression of *Arc* mRNA would be due to STFP recall procedures, while nuclear expression would be due to FC/FCbP recall procedures, with cells showing dual activation having been activated at both timeframes (see Fig. 1; also, see “Tissue analysis” for details on tissue treatment and processing).

### Procedures

#### Habituation and food restriction

All habituation took place just prior to the first day of experimental procedures. Habituation consisted of each cage of rats being moved into the room in which all STFP experimental procedures would take place and being allowed to habituate to the room for 15 min. During this period, each rat was picked up and handled by the experimenter that would be running behavior for 2 min to habituate them to handling and that individual. All habituation procedure took place in a dark room under red light, and all rats received 4 days of habituation. Food restriction began the day before habituation began and persisted to the end of the experiment. At the start of food restriction, the food pellets that all subjects had been eating were removed from the cage. Subsequently, all cages were given daily ad libitum access to a hanging jar full of plain, powdered Purina 5LL2 diet in their home-cage for 1 h. Rats were weighed daily starting at the beginning of food restriction until the experiment was over to ensure no unusual loss in weight.

### Behavioral scoring

All behavioral scoring for this experiment was completed using the Behavioral Observation Research Interactive Software (BORIS)^60^.

#### Fear conditioning by-proxy social contact scoring

Past research from our lab has indicated that there is a strong relationship between the amount of fear displayed by observers at the long-term memory test and the time spent interacting with their Demonstrator during the CS in males^13^ and after the CS in females^14,26^. As such, videos of the social acquisition phase of fear-conditioning by proxy were scored for social interaction between the Observer and Demonstrator for each 20 s period during the CS presentation and the 20 s period immediately following each CS presentation to provide a secondary index of fear acquisition. Social contact was scored when Observer and Demonstrator rats made contact other than in passing during the cue period (during CS contact) or in the 20 s following the CS (post CS contact). The percentage of each score period spent in contact with the Demonstrator was calculated. Data for percent contact during the cue period for males and data for the percent contact immediately following the cue period for females was pulled and combined into a single “relevant contact” measure to be used in all final statistical analyses.

#### Choice test scoring

Videos of the choice test to initiate recall of a socially transmitted food preference were scored for the amount of time that a given rat spent interacting with a food cup based on whether it contained the demonstrated/already consumed diet (diet Cin) or the novel diet (diet Co). This was done as a potential secondary measure of food preference, as we anticipated that due to the choice test being abnormally short (10 min) by necessity that we might be unable to detect preferences based on amount eaten alone. Interaction with the food cup was scored for whenever a rat was physically in contact with and not actively moving away from the cup (i.e., front paws in contact with the jar, head inside jar, climbing on top of the jar, or actively eating from the jar). For statistical analysis, we calculated the percent of time spent interacting with a cup containing a given diet based on the total amount of time spent interacting with either cup (e.g., for diet Cin, Percent time = Time_Diet Cin_/(Time_Diet Co_ + Time_Diet Cin_)). The full 10-min choice test session was scored for all rats that underwent the choice test with the exception of one rat whose video was unavailable due to recording equipment failure.

### Tissue analysis

To minimize degradation of mRNA by ribonuclease (RNase), all equipment and surfaces used during brain preparation and processing were sanitized regularly with either RNase AWAY™ (Thermo Scientific; Waltham, MA, USA) or RNAseZap™ (Ambion; Grand Island, NY, USA).

#### Brain preparation

Immediately following euthanasia, subjects were perfused intracardially using a 4% paraformaldehyde (PFA) solution. Brains were then removed and submerged in 4% PFA to allow post-fixation for 24–48 h. Once post-fixation was complete, brains were transferred to a solution of 30% sucrose in phosphate buffered saline for cryoprotection. Once brains had sunk to the bottom of the vial, indicating sufficient sucrose uptake for cryoprotection, they were flash frozen in powdered dry ice and moved to a – 80 °C freezer for storage until sectioning. Brains were then sectioned coronally on a sliding microtome at 30 µm thickness into six series (so subsequent sections in a single series were 180 µm apart) and immediately mounted and allowed to air dry before being placed in a vacuum chamber with humidity sponges where they were left to dry fully for 24 h. Only hippocampal sections (approximately − 3.2 to − 5.2 from bregma) or prefrontal regions (approximately + 3.7 to + 1.4 from bregma) containing the areas of interest were sectioned and processed. Mounted sections were then placed in a sealed RNase-free slide box and stored at – 80 °C until processing.

#### Tissue processing

All procedures were modified from the protocols used in Lee et al.^61^ and Petrovich et al.^62^. Prior to tissue processing, a cRNA probe for *Arc* mRNA was constructed starting with a plasmid containing a full-length cDNA (~ 3.0 kbp) of the *Arc* transcript. To create the probe, the DNA was first cut by mixing the plasmid with a 10 × digestion buffer (NEBuffer; Biolabs; Ipswich, MA, USA), a 10 × EcoRI restriction enzyme (Biolabs), and purified nuclease free water before being incubated at 37 °C for 2 h. Proper cutting of the DNA was verified using electrophoresis, after which the DNA was purified overnight in ethanol. Following purification, the DNA pellet was spun out in a centrifuge, washed in EtOH, fully dried, and resuspended in a TE buffer. To verify that the DNA was properly linearized, calculate *Arc* concentration, and check that no contaminants were present, a sample of the DNA was tested via spectrophotometry (Nanodrop Lite; Thermo Scientific, Waltham, MA, USA). The Digoxigenin (DIG) labelled probe was transcribed by combining the linearized DNA with RNase free water, a 10 × transcription buffer (Ambion), RNAse block (Ambion), DIG RNA labelling mix (Roche Applied Science; Indianapolis, IN, USA), and a T7 RNA polymerase (Ambion) before incubating the solution at 37 °C for 2 h. Finally, the probe was diluted in nuclease free water and purified in a mini Quick-Spin column (Roche).

Once the cRNA probe had been constructed, slides containing tissue from the male rats were submerged for 40 min in a 4% PFA solution to increase tissue integrity throughout in situ processing. Tissue from female rats were processed without this PFA wash. Slides were then washed and incubated in a proteinase K (PK) buffer at 37 °C before being treated with a 0.5% acetic anhydride/1.5% triethanolamine solution containing glacial acetic acid for permeabilization. Slides were then washed in a saline-sodium citrate (SSC) buffer before being dehydrated by submersion in ascending concentrations of ethanol and air dried. Finally, each slide was covered in 300 µL of a hybridization buffer containing yeast tRNA (Invitrogen; Carlsbad, CA, USA), salmon sperm DNA (Ambion), dithiothreitol (Sigma; St. Louis, MO, USA), and the cRNA probe (1:100). Each slide was then cover slipped and sealed around the edges with DPX mountant (Electron Microscopy Sciences; Hatfield, PA, USA) before being incubated in the hybridization solution for 20 h at 60 °C. Notably, the coverslip protected the tissue from any direct contact with the DPX mountant, so later removal of the hardened DPX caused no damage to the tissue.

Once hybridization was complete, cover slips were carefully removed, and slides were incubated in a 4xSSC buffer mixed with sodium thiosulfate (ST) at 60 °C for an hour before being treated with an ethylenediaminetetraacetic acid-based solution to inhibit RNAse activity at 37ºC. Following this, slides were washed in descending concentration of SSC solution mixed with ST again at 60 °C. Tissue was then washed in a detergent solution (Tween20) before being stained with the PerkinElmer TSA Fluorescein system (NEL701001KT; PerkinElmer, Waltham, MA, USA). Slides were placed in a humid chamber and treated with blocking buffer followed by an anti-DIG-HRP conjugate for 2 h. Slides were then briefly washed in the detergent solution before being returned to a dark humid chamber and coated with a solution containing fluroscein tyramide reagent (FITC) and allowed to sit for 30 min. Finally, slides were washed, allowed to air dry, and cover slipped with a mountant containing the nuclear stain 4′,6-diamidino-2-phenylinodole (DAPI) (Vectashield; Vector Lab, Burlingame, CA, USA). Slides were stored in the dark at – 20 °C until imaging.

#### Imaging

All imaging was completed using an Axio Scope A1 microscope (Zeiss; Thornwood, NY, USA). Regions of interest were identified via DAPI staining using a 10 × objective with the assistance of the Paxinos and Watson brain atlas^63^ and then imaged under a 40 × objective (actual magnification ~ 900 ×). Images were taken for both DAPI and FITC stains and later colorized and merged automatically using a custom macro in the ImageJ software with FIJI (NIH, Bethesda, MD). Due to tissue degradation occurring over the course of in situ not all sections or areas of potential interest were viable. As such, images were not able to be z-stacked reliably and, instead, were taken on a single plane. The following regions were imaged and counted: the prelimbic cortex (+ 3.72 to + 2.52 from bregma), the infralimbic cortex (+ 3.52 to + 2.2 from bregma), the lateral (+ 3.72 to + 3.2 from bregma) and ventral (+ 3.72 to + 3.0 from bregma) orbitofrontal cortex, the CG1 region of the anterior cingulate cortex (+ 3.72 to + 2.52 from bregma), and the CA3 region of the ventral hippocampus (− 4.3 to − 4.8 from bregma) (Fig. S6). Though the amygdalar nuclei were also of particular interest for their well-established role in fear learning, the aforementioned tissue damage tended to be particularly severe in this area. As such, we were not able to obtain a sample size large enough to include that region (a minimum of 6 viable images/region was required for a rat to be included in the statistical analysis of a given area).

Counts were completed region by region and all image files were assigned a random numerical code to blind the experimenter completing the counts from any details concerning the image at the time of counting. All cell counts were taken in ImageJ with the FIJI package and were made using the cell counting tool. Cells were counted for nuclear and cytoplasmic *Arc* mRNA expression separately and cells showing overlapping expression were counted as dual expressing. The final counts for nuclear *Arc* expressing and cytoplasmic *Arc* expressing cells included only those cells expressing in only that region (i.e., did not include dual expressing cells). Full counts for DAPI stained cells were taken and the percent of cells showing expression in each given area was calculated followed by the average percent of cells showing each type of activation in individual rats. To prevent the scores of rats with larger numbers of images from having a disproportionate effect on our statistics and to prevent an inflation of sample size only these averages were used in our final analysis.

#### Statistical analysis overview

All statistical analyses were completed using the R coding software. The full code is freely available to view at our data repository at https://dataverse.tdl.org/dataverse/MonfilsFearMemoryLab. Unless otherwise stated, the cutoff for a test to be considered statistically significant was set to p < 0.05. All of our *Arc* results, unless otherwise mentioned, were tested for significance using a series of two-way ANOVAs (type 2) containing sex and condition as between subject variables (Sex and Condition) with an individual ANOVAs run for each area of expression (nucleus, cytoplasm, and dual). Similarly, a series of one-way ANOVAs were run with a combined variable containing the food task (diet Cin only or Choice test for Demonstrators and Observers; plain chow only for all Controls) for each area of expression. Sex was not included as a secondary variable as the relatively low number of female rats made sample sizes too small for certain condition/food task combinations. When ANOVA assumptions were violated, data were transformed using either a log(y + 1) function or by taking the inverse square root. As these transforms did not always succeed in bringing ANOVAs in line with assumptions, Kruskal-Wallace tests were performed on datasets where transforms were not effective. Pairwise t-tests were performed for post-hoc analyses against a Bonferroni-corrected alpha value when ANOVAs indicated a significant effect of condition (α = 0.017) or a significant sex and condition interaction (α = 0.008; conditions tested against each other within each sex only) with between-group effect sizes calculated using Cohen’s d. To provide a better gauge of variability for our smaller group sizes, the MS_Error_ obtained from our ANOVA was used in the denominator of post-hoc t-tests. Effect sizes for ANOVAs were calculated using the standard partial η^2^ formula and for Kruskal-Wallace tests using the formula η^2^_H_ = (H − k + 1)/(n – k). For simplicity of data presentation, unless the addition of the food task grouping variable resulted in a significant effect or unless a significant contribution of sex was detected all data were presented graphically split up by area of expression and overall experimental condition only. Any rats that had fewer than 6 viable images counted in a given brain region were excluded from the analysis for that area.

Bivariate correlations were calculated for Observer and Demonstrator rats to assess potential relationships between behavioral measures and *Arc* cell counts for expression occurring at appropriate timepoints (e.g., cytoplasmic *Arc* counts for percent of cinnamon eaten). Pearson’s correlation coefficients were used in the event that no outliers in either dataset were detected with a Grubbs test. If outliers were detected, Spearman’s correlation coefficient was used instead. To gauge whether a relationship between social learning and *Arc* in dual expressing cells in Observers, an overall metric of social learning—referred to from here on out as the social learning metric (SLM)—was calculated by taking the mean of the standardized scores (z-score) for the percentage of total eaten that was the demonstrated food and the percentage of time spent in contact with the Demonstrator during the FCbP social learning phase during the CS presentation (males) or after the CS presentation (females). Notably, percent freezing to the cue on the final day was not used for Observer rats because our results and the results of our follow up experiment (see Supplementary Materials) indicated that the conditions of our behavioral testing procedure resulted in some freezing behavior even in Control rats—at least in males—and, as such, might not be the best gauge of the strength of the socially acquired fear response. As such, given our past findings that interactions with the Demonstrator during or after the CS (depending on sex) highly predicted later freezing to the cue^12–14^, interaction with the Demonstrator at the sex appropriate timepoint was tested for correlations against nuclear *Arc* activity rather than freezing to the cue on the final day for Observer rats. For Demonstrators, a similar metric was calculated based on standardized freezing to the cue on the final day and the percent of total eaten that was the familiar diet (Diet Cin) and checked against dual *Arc* activity. To correct for the multiple tests run on each behavioral dataset (6, for each brain region), the critical p-value for correlations was Bonferroni adjusted to 0.0083.

### ARRIVE compliance

The studies reported in this manuscript were conducted in accordance with ARRIVE guidelines.

## Supplementary Information

Supplementary Information.

## Data Availability

All code and raw data files are available in The Monfils Lab repository, housed in the Texas Data Repository in Dataverse (https://dataverse.tdl.org/dataverse/MonfilsFearMemoryLab). All other materials are available by request to the authors (MHM or LAA).
