# Supplementary material for: Patterns of Arc mRNA expression in the rat brain following dual recall of fear- and reward-based socially acquired information
Source: Sci Rep. 2023 Feb 10;13:2429. doi: 10.1038/s41598-023-29609-6 (PMC9918527; doi:10.1038/s41598-023-29609-6)
Supplement: Supplementary file 1 — Supplementary Information. [file 41598_2023_29609_MOESM1_ESM.docx]

**Supplementary –**

**Patterns of Arc mRNA expression in the rat brain following dual recall of fear- and reward-based socially acquired information**

Laura A. Agee, Emily N. Hilz, Dohyun Jun, Victoria Nemchek, Hongjoo J. Lee, and Marie-H. Monfils

**Supplementary Methods and Results** p2-p13

**Supplementary Figures and Tables** p14-p24

**Contact Information:**

Marie-H. Monfils

[marie.monfils@gmail.com](mailto:marie.monfils@gmail.com)

The University of Texas at Austin

Department of Psychology

108 E. Dean Keeton Stop A8000

Austin, TX 78712-1043

**Supplementary Methods and Results**

**Dominance Assessments**

*Play behavior dominance assessment*

A day prior to play dominance assessments, all males were moved to single housing to promote social play behavior. Following a 24-hour isolation period, individuals from each triad were moved to a large plastic bin (50.5 cm × 39.4 cm × 37.5 cm) with woodchip bedding and a camera mounted overhead to record behavior. Rats were allowed to interact for 15 minutes before being removed from the box and returned to single housing. This was repeated for 3 sessions, after which rats were returned to their triads and left undisturbed until the start of the milk competition dominance assessment. Behavior was scored as described below, and rats in Cohort 2 were assigned to one of three dominance ranks based on their behavior as following with past research on dominance hierarchies in rats ^1^: Dominant, Subordinate 1, or Subordinate 2. Male rats in cohort 1 were randomly assigned condition regardless of dominance rank. As described in Jones & Monfils (2016), dominant rats were the rats that received most nape contact (i.e., play initiations), while subordinate 1 was the rat that initiated the dominant the most, and subordinate 2 tended to be avoidant. While all Cohort 1 males were used, male triads in Cohort 2 that did not show dominance hierarchies were removed from the study and used in other experiments. Analysis of play behavior dominance included only Cohort 2 rats.

*Milk competition dominance assessment*

In order to validate dominance assignments made using the play behavior assessment, we recorded and scored the behavior of male rats allowed access to a desired resource (sweetened milk solution), a dominance assessment that our lab previously found to be effective (Jones & Monfils, 2016). The milk solution used in this dominance assessment was a mixture of 2/3 tap water and 1/3 sweetened condensed milk (Eagle^TM^) stored in a 2 oz glass jar filled to the top with the solution. Prior to running the dominance assessment, rats from all male triads were moved to single-housing and given access to a full jar of the milk for 5-hours to ensure that each individual rat had the opportunity to overcome their neophobia of the milk solution. Following this, rats were returned to their triads and allowed access to a full jar of the milk solution as a group daily for four days. In order to assure that rats would be motivated to drink, food hoppers were removed from all triads 12 hours before the milk was introduced. Following the 3-hour milk access period, hoppers were returned until removal time for the next day of habituation.

Once habituation to the milk solution had been completed, triads were run through the formal dominance assessment. As during habituation, food hoppers were removed 12 hours before the start of assessment to promote competition. 2 oz glasses were filled with to the top with the milk solution and secured with adhesive strips to the bottom of a large plastic bin (50.5 cm × 39.4 cm × 37.5 cm) with woodchip bedding and portable cameras were mounted above the box for an over-the-head view of all behavior. Rats were placed in the bin and allowed access for either 12 minutes (Cohort 1) or 10 minutes (Cohort 2) before being removed and returned to their triads. While only two sessions of the competition were run for our Cohort 1 males, three sessions were run for Cohort 2 in an attempt to obtain clearer dominance hierarchies.

*Play dominance scoring*

Behavior was scored for the full play session, with both offensive play behaviors (i.e., play initiations or attacks) and defensive play behaviors (i.e., response to play initiations, specifically nape contact) being scored (see Pellis & Pellis, 1991, 1993; Jones & Monfils, 2016). The following offensive play behaviors were scored: (1) Nape contact, contact of a rat’s snout with the nape of another rat and (2) Boxing, which occurred when rats reared and punched at each other with their front legs. The defensive behaviors scored for were: (1) Counter, in which the attacked rat turns to face the attacking conspecific to launch an attack of their own; (2) Evasion, in which the attacked rat flees from the attacker; (3) Full rotation, in which the target rotates fully into a supine position; (4) Half rotation, in which the targeted rat responds to the attack by shifting their body laterally to break contact without fully losing their feet; (5) No response, in which the target either freezes or carries on at a normal pace in response to attack. The identity of both the initiating rat and their target was noted for every instance of play behavior. Across all sessions, the total nape contacts received for each individual rat was tallied and divided by the total number of nape contact initiated in the cage to determine the percent of contacts each rat had received. If a rat had received a disproportionate amount of contact (>40%) they were deemed the dominant rat.

*Milk competition scoring*

Behavior for milk competition began to be scored as soon as all rats were in the bin and the experimenter had exited the footage. Behavior was scored in 1-minute bins for 10-12 minutes. The duration of each subject drinking from or monopolizing the milk jar (i.e., drinking from or having paws/body on the jar and preventing the other rats’ access) was scored for each 1-minute interval. To calculate percent monopolization of the resource, the total time all rats spent drinking in each bin was summed and the time spent drinking for individual rats was divided by that value. The amount of time spent drinking was then plotted based on play behavior dominance assignments for all rats.

*Dominance tests results*

As only Cohort 2 rats were assigned conditions based on dominance rank, Cohort 1 males were not included in these analyses, resulting in data from nine triads (n = 27 rats) being included. To verify our dominance assignments, we ran a two-way ANOVA (Type 2) with the percent of total nape contacts in the cage received as the dependent variable and engaging rat rank and responding rat rank as independent variables. The interaction had to be tested separated using a one-way ANOVA. We found an overall effect of both engaging (F_(2,49)_ = 16.409, p < 0.0001) and responding (F_(2,49)_ = 19.490, p < 0.0001) rank and an interaction between the two (F_(5,48)_ = 9.84, p < 0.0001). A post-hoc Tukey HSD found that, as expected, dominant (p = 0.0082) and S1 (p = 0.043) were significantly more likely to engage than S2 rats, and dominants were more likely to be the responder when compared to both S1 (p = 0.0031) and S2 (p = 0.002) rats. S1 rats were also significantly more likely to contact the dominant rat than the S2 rat (p = 0.00031). We also examined the percent of times a rat responded to a nape contact with a counter, a behavior that has previously been found to be more likely in dominant rats ^3^. Differences in likelihood of counter response was tested using a series of Kruskal-Wallace tests due to violations of ANOVA assumptions. We found that while there was no detected effect of engaging rank (H_2_ = 3.23, p = 0.199) there was a significant effect of responding rank (H_2_ = 7.92, p = 0.0191) and a post-hoc Dunns test with Holm’s p-adjustment found that dominant assigned rats did counter significantly more than rats assigned to the S1 condition (p = 0.0162) but not rats assigned to the S2 condition (p = 0.156). A mixed-effects ANOVA run to examine performance during the milk dominance assessment with percent of time monopolizing the milk cup as the dependent variable, assigned rank as the between-subjects variable, and minute of scoring as the within-subjects variable. We found a significant overall effect of rank (F_(2,24)_ = 4.83, p = 0.0172) but no effect of minute (F_(9,216)_ = 0, p > 0.99) and no interaction between the two (F_(18,216)_ = 1.189, p = 0.272). Post-hoc pairwise comparisons across the various ranks averaged across minute found that S1 ranks rats spent significantly more time monopolizing the milk cup than S2 rats (p = 0.0165) and dominant rats also trended in that direction (p = 0.09), but there was no significant difference between dominant rats and S1 rats (p = 0.713) (see *Fig S1*). Notably, these results are counter to earlier findings from our lab ^2^, which might be attributable to differences in the container used to hold the milk during testing as the lid of our container was slightly wider (4.45 cm Diameter vs. 3.75 cm diameter) making the milk more easily accessible.

**Fear Conditioning by-Proxy in Dark Cycle with Control Cue Exposure**

Past behavioral experiments run in our lab using the fear conditioning by-proxy (FCbP) behavioral paradigm had rats run during their light cycle for all phases of the procedure and did not involve control rats receiving CS exposure prior to long-term memory testing (Bruchey, Jones, & Monfils, 2010; Jones et al., 2014; Jones & Monfils, 2016; Agee, Jones, & Monfils, 2019). In order to determine whether the lack of difference in freezing between our control and observer rats on the final day of experimentation in the main experiment was due to the methodological changes that we had made in the fear conditioning by-proxy (FCbP) for the primary experiment, a follow up experiment partially replicating our FCbP procedures with a full long-term memory (LTM) test was run.

*Subjects and scoring*

Subjects were 24 male Sprague-Dawley rats (n = 8/condition) that had been housed in their triad for a month and had previously been run through a novel object experiment that took place in the conditioning chambers. Therefore, all rats were familiar with the conditioning chambers but had only ever interacted with a novel object within the chamber. One rat from each triad was randomly assigned to the control, observer, and demonstrator condition. Following the experimental procedure, videos of the long-term memory test were scored for freezing to just prior to the first cue (Pre-CS freezing) and freezing to all three subsequent cues using the same scoring method as described in the main paper.

*Methodology*

The FCbP procedure was run exactly as described in the main text with the following exceptions: (1) all rats were given three exposures to the conditioned stimulus (CS) during the long-term memory test on the final day, (2) control rats were run through cue exposure on the same day as FCbP acquisition took place for observer rats, and (3) the long-term memory test took place 24 hours following FCbP training/cue exposure rather than 48 hours after FCbP training/cue exposure, and (4) demonstrators and observers did not go through the social transmission of food preference (STFP) procedure after the FCbP interaction (see *Fig S2*). Notably, to prevent the behavior of observer and demonstrators (specifically, potential olfactory or auditory cues) from influencing controls during cue exposure, all control rats were run prior to observers and demonstrators being placed in the chamber.

*Results*

A two-way mixed ANOVA was run on the percent of time freezing during or just prior to CS presentation with cue period (pre-CS, CS1, CS2, and CS3) as the within-subjects variable and experimental condition as the between-subjects variable. A significant overall effect of condition (F_(2,21)_ = 7.588, p = 0.0033) and cue (F_(3,63)_ = 10.68, p < 0.0001) but no interaction between the two (F_(6,63)_ = 0.73, p = 0.62). Post-hoc Dunns tests with Holm’s adjusted p-values for multiple comparisons found that demonstrators displayed significantly higher freezing than observers (p = 0.0018) and controls (p < 0.00001), but observers did not freeze significantly more than controls (p = 0.11). They also confirmed that the only significant differences in freezing between cue periods was between the Pre-CS period and the CS1 (p = 0.014) and CS2 (p = 0.00563) periods, though the difference between pre-CS and CS3 was only nearing significance after correction (p = 0.078) (see *Fig S3a*). A one-way ANOVA was also run on the percent freezing to cue averaged across the three CS presentations with condition as the between-subjects variable. While the ANOVA found an overall effect of experimental condition (F_(2,21)_ = 16.95, p < 0.0001), a post-hoc Tukey HSD again found that this difference was significant between demonstrators and observers (p = 0.0009) and demonstrators and controls (p < 0.0001) but not between controls and observers (p = 0.428) (see *Fig S3b*). These results back up our interpretation of the lack of difference in freezing between our observers and controls as likely being the result of our methodological changes to the FCbP paradigm.

**Effect of STFP and FCbP on freezing response in directly conditioned rats**

To determine whether the unusually low freezing that was documented in our demonstrator rats at the final long-term fear memory test was: (1) replicable and (2) the result of social component of the behavioral procedures that demonstrators had undergone during the second day of the experiment, a new set of rats were run through iterations of the behavioral procedure with varying amounts of social interaction.

*Subjects*

Subjects were 48 male Sprague-Dawley rats that were ~6 weeks of age at arrival. Rats were housed in triads immediately after arrival. Testing did not start until at least a month after arrival to ensure that the rats in each triad had sufficient time to form social bonds. Rats were left undisturbed until four days prior to the start of behavioral testing, at which point they were food restricted and habituated to handling procedures/testing areas as described in the main text. 16 rats served as familiar social stimulation only, resulting in an n = 8 group size for each of the four experimental conditions.

*Methodology*

(See *Fig S4* for a graphical overview of the behavioral design)

Rats in each triad were assigned to one of three conditions: (1) Demonstrator 1 (Dem1), (2) Demonstrator 2 (Dem2) or (3) Observer. As we were only interested in the behavior of the demonstrator (i.e., the directly fear conditioned rat) rats in this case, observer rats served purely as a familiar rat to provide the social component of the behavioral paradigm. On day 1 of the experiment, both demonstrators went through fear conditioning exactly as described in the main text. On day 2, demonstrators were re-exposed to the CS 3 times either in the presence of their paired observer or alone before either being transported to single housing or being given access to cinnamon flavored powdered chow for 1 hr, after which they were allowed to interact with their triad’s observer for 30 minutes. This results in there being four possible experimental conditions: (1) Recall with cage mate and STFP (RC+STFP), (2) Recall with cage mate only (RC), (3) Recall alone only (RA), and (4) Recall alone and STFP (RA+STFP). The recall procedure was the same protocol as what was used for the FCbP in the primary experiment. As Dem1 and Dem2 were run at the same time, in triads in which one demonstrator was assigned to the RC+STFP condition the other demonstrator would always be assigned to the RA condition. Similarly, in cages in which one demonstrator was assigned to the RC condition, the other was always assigned to the RA+STFP condition. Following day 2 behavioral procedures, observer rats were euthanized and demonstrators were moved to single housing.

48 hours later, on the final day of behavioral procedures, all demonstrators were given access to cinnamon and cocoa chow and allowed 10 minutes to consume as much of either as they pleased. After this, both demonstrators were returned to their home cage for 10 minutes before being returned to the conditioning chamber and allowed to habituate for 5 minutes before being given three 20 second CS presentations. Three CSs were given rather than the single presentation in the primary experiment because we deemed it more important to be able to thoroughly investigate their behavioral response than to ensure clean *Arc* expression. Demonstrators were, however, perfused as described in the main text and brains were removed, cryoprotected, and flash-frozen in case the behavioral results warranted tissue analysis.

*Results*

A two-way mixed ANOVA was run on the percent freezing to the CS on recall day 2 with cue period (pre-CS, CS1, CS2, CS3) as the within-subjects variable and experimental condition as the between-subjects variable. A significant effect of cue period (F_(3,84)_ = 17.49, p < 0.0001), but not experimental condition (F_(3,28)_ = 0.78, p = 0.52), or any interaction between the two (F_(6,56)_ = 1.14, p = 0.35) was detected. A post-hoc Dunns test with Holm’s correction confirmed the effect of cue was driven by freezing at the pre-CS period (p < 0.001 vs freezing during all CSs) (see *Fig S5a*). A set of planned pairwise comparisons (two-way t-tests with Holms adjusted p-values) were run to check for differences in freezing to CS1 only between the RC+STFP condition and each other condition. No significant differences were detected between rats in the RC+STFP condition and rats in any of the other conditions (all p > 0.1). Finally, a two-way factorial ANOVA run on the average percent freezing during each CS presentations found no significant effect of recall condition (F_(1,28)_ = 0.06, p = 0.81) or STFP condition (F_(1,28)_ = 0.96, p = 0.34) and no interaction between the two (F_(1,28)_ = 0.46, p = 0.51) (see *Fig S5b*). While the freezing to CS1 by the rats that were assigned to the behavioral condition that was most similar what the demonstrators had undergone in the main experiment (RC+STFP) (n = 8, mean = 43.2, SD = 24.0) was higher than the freezing observed in our demonstrators at the final long-term memory test of our primary experiment (n = 30, mean = 25.2, SD = 18.5), this difference did not quite reach significance when the scores of the demonstrators were compared against each other with a two-sample t-test (t_9.3_ = -1.97, p = 0.079). It is possible, if unlikely, that adding more rats to the RC+STFP condition would have resulted in their CS1 freezing scores regressing towards the mean of the freezing of our demonstrators in the main experiment. In interpreting these results, it is also important to note that the social relationship between the observers and demonstrators in this experiment were quite different from the relationship between observers and demonstrators in the original experiment (unrelated 1-month cage mates vs. siblings housed since weaning) and this may explain the relatively lower freezing observed in the demonstrators in our primary experiment.

**Supplementary References**

Agee, L. A., Jones, C. E. & Monfils, M.-H. Differing effects of familiarity/kinship in the social transmission of fear associations and food preferences in rats. *Anim. Cogn*. **22**, 1013–1026 (2019).

Bruchey, A. K., Jones, C. E. & Monfils, M.-H. Fear conditioning by-proxy: Social transmission of fear during memory retrieval. *Behav. Brain Res*. **214**, 80–84 (2010).

Jones CE, Monfils M-H. Dominance status predicts social fear transmission in laboratory rats. *Anim Cogn.* **19**(6), 1051–69 (2016).

Jones, C. E., Riha, P. D., Gore, A. C. & Monfils, M.-H. Social transmission of Pavlovian fear: fear-conditioning by-proxy in related female rats. *Anim. Cogn*. **17**, 827–834 (2014).

Pellis SM, Pellis VC. Role reversal changes during the ontogeny of play fighting in male rats: Attack vs. defense. *Aggressive Behavior.* **17**(3),179–89 (1991).

Pellis SM, Pellis VC, McKenna MM. Some subordinates are more equal than others: Play fighting amongst adult subordinate male rats. *Aggressive Behavior*. **19**(5), 385–93 (1993).


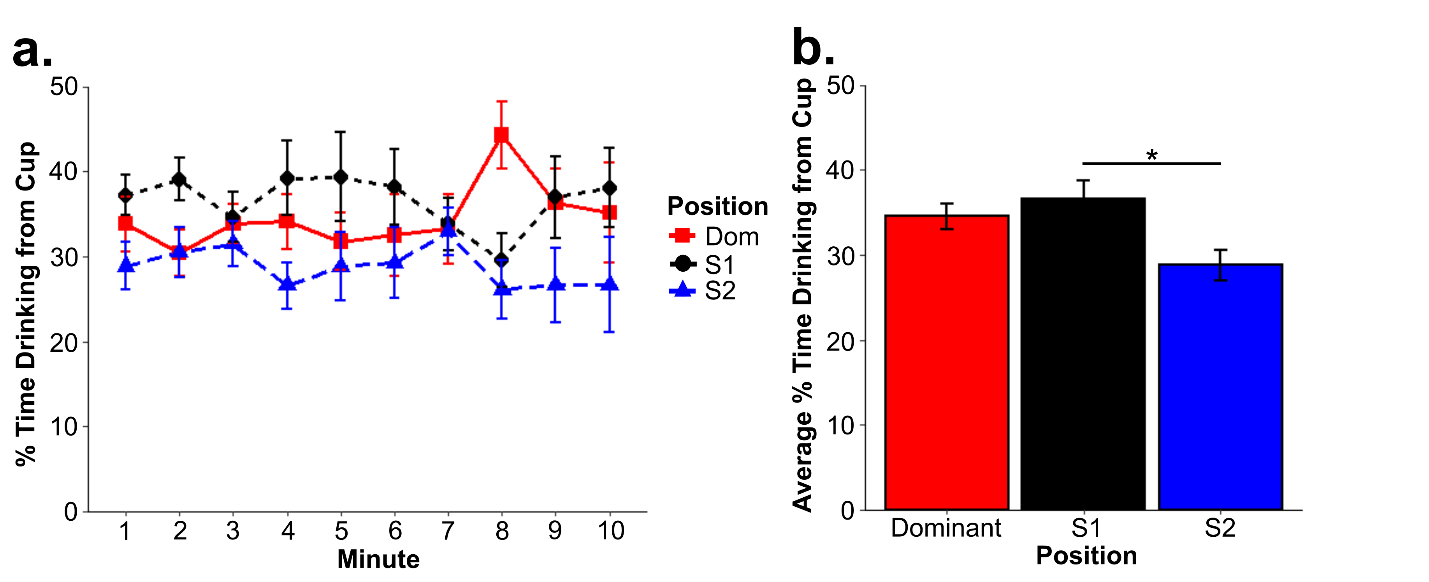


**Figure S1. Milk dominance test results.** The above figures show the average percent of total time that rats assigned a given rank spent monopolizing the milk cup (a) across the first ten minutes of the dominance test and (b) averaged across each minute by rank. **p < 0.05*



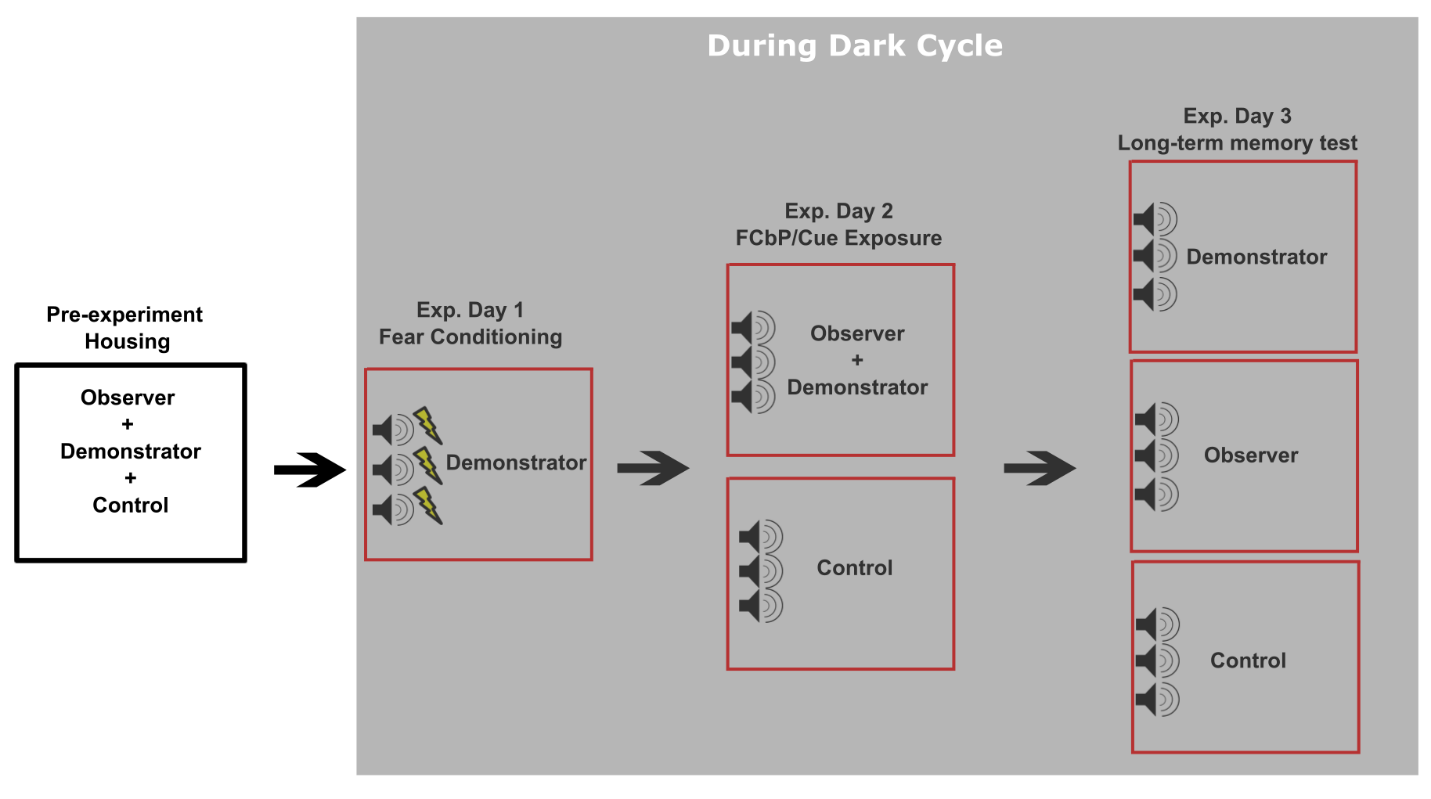


**Figure S2. Dark cycle fear conditioning by-proxy with control cue exposure***.* The above figure outlines that procedure for our follow-up experiment examining long-term freezing response to an auditory CS in rats that had undergone FCbP during their dark cycle when compared to control rats that had received cue-exposure alone.


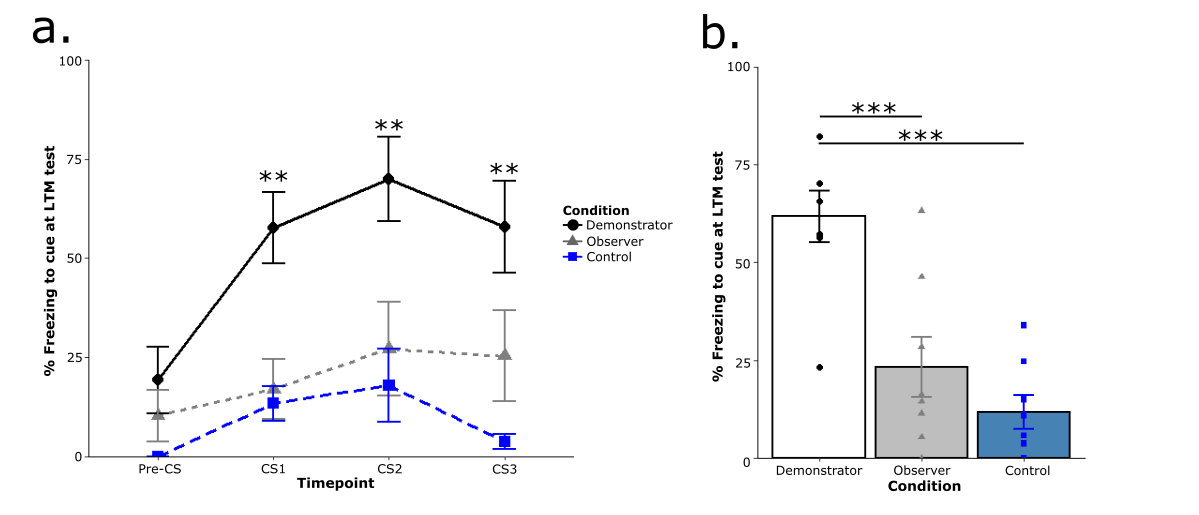


**Figure S3. Dark cycle FCbP results***.* (a) While demonstrators showed significantly higher freezing to the CS at all presentations as compared to observers and controls, observers did not freeze significantly more than controls and (b) this remained true when percent freezing was averaged across the three CSs.

***p < 0.001, ***p < 0.0001*


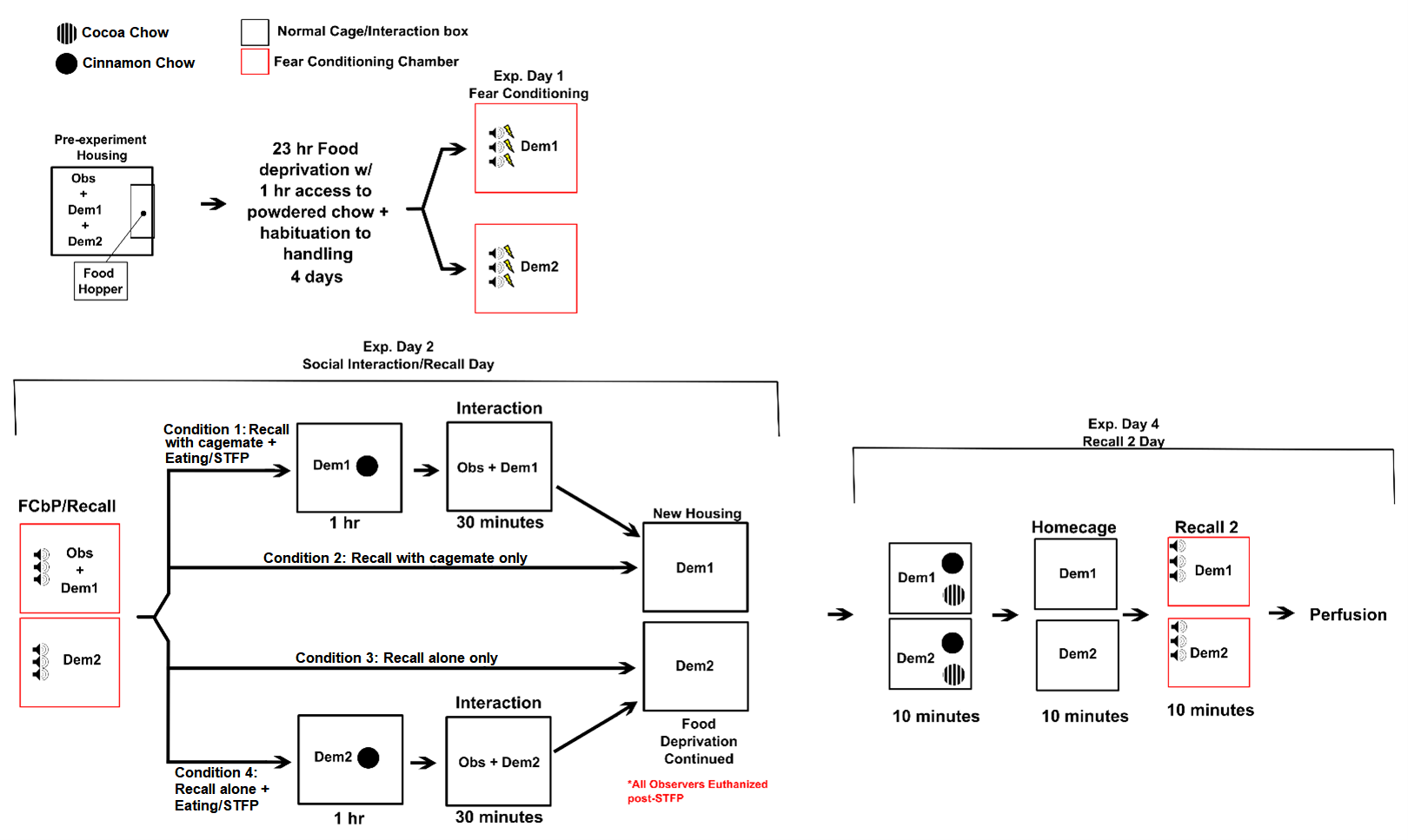


**Figure S4. Effect of FCbP and STFP behavioral protocols on directly conditioned demonstrators.** This figure displays the experimental design of our follow-up experiment that was run to try and replicate the reduced freezing observed in our demonstrator rats in the primary experiment.


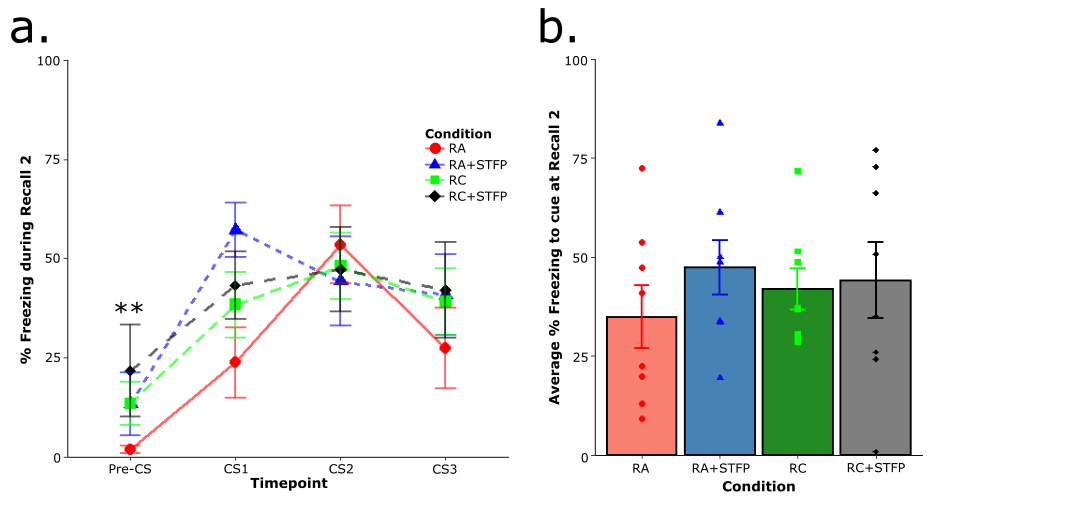


**Figure S5. FCbP+STFP effect on directly conditioned rats - Results***.* (a) There was no difference in freezing behavior between any of the experimental conditions (Recall alone [RA], Recall alone with STFP [RA+STFP], Recall with cagemate [RC], and Recall with cagemate and STFP [RC+STF]) and (b) this remained true when freezing was averaged across all three cues.


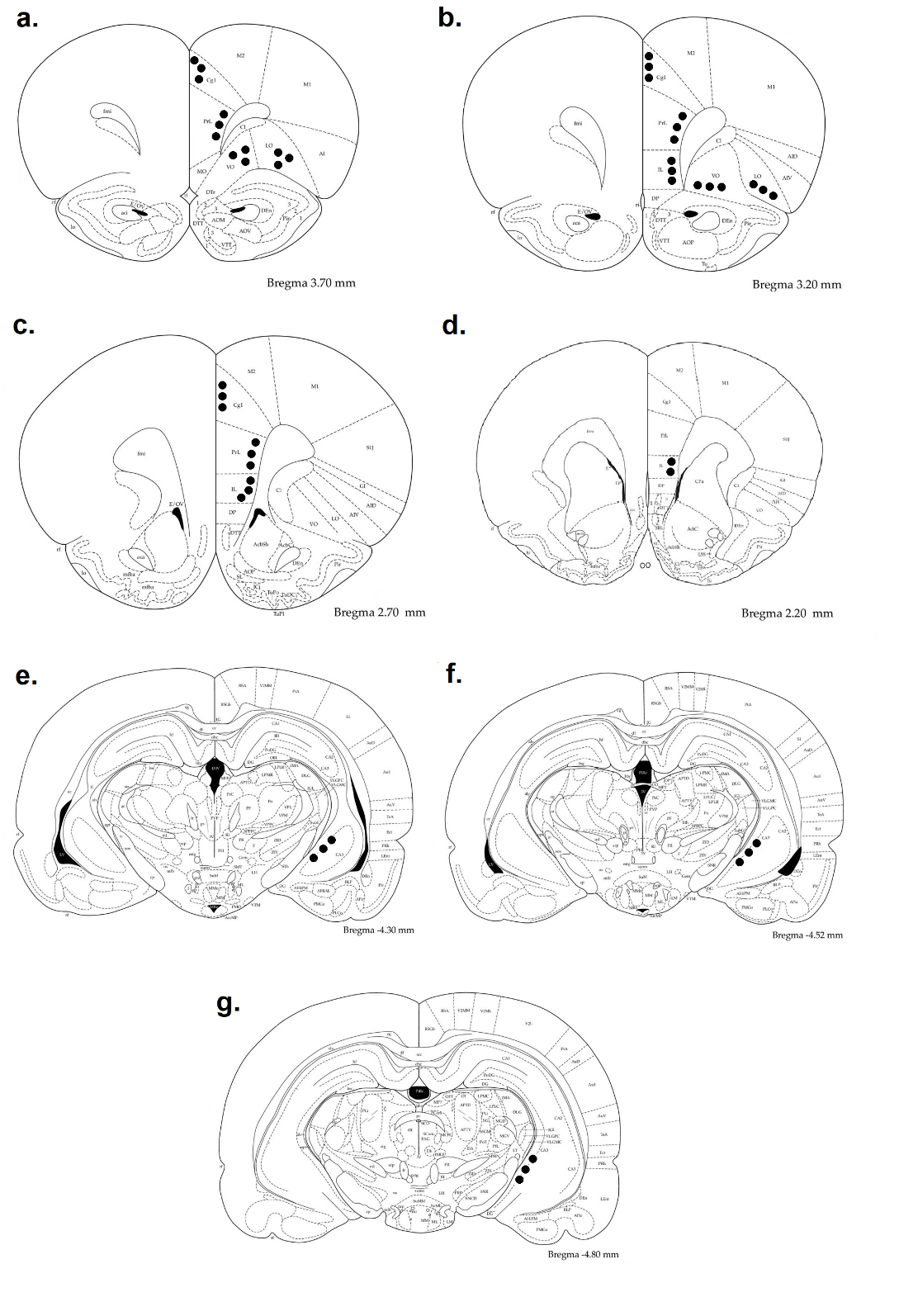


**Figure S6. Representation of sampled areas.** Images of coronal rat brain sections adapted from Paxinos and Watson (2006). The blacked-out circles indicate the approximate areas sampled from each plane for (a-d) the prelimbic, infralimbic, CG1 region of the anterior cingulate cortex, and the ventral and lateral orbitofrontal cortices, and (e-g) the CA3 region of the ventral hippocampus.

| Table S1. Main Effects of Sex on *Arc* Expression | | | | | | |
| --- | --- | --- | --- | --- | --- | --- |
| Cell Area | Nucleus | | Cytoplasm | | Dual | |
| Brain Region | Statistical value | p-value | Statistical value | p-value | Statistical value | p-value |
| lOFC | F_(1,57)_ = 0.31 | 0.581 | F_(1,57)_ = 0.01 | 0.928 | F_(1,57)_ = 6.18 | 0.016* |
| vOFC | F_(1,64)_ = 4.85 | 0.031* | F_(1,64)_ = 0.01 | 0.905 | F_(1,64)_ = 1.58 | 0.214 |
| ACC | F_(1,66)_ = 0.07 | 0.788 | F_(1,66)_ = 3.22 | 0.078+ | F_(1,66)_ = 15.93 | < 0.001*** |
| Ifl | F_(1,73)_ = 18.05 | < 0.001*** | H_1_ < 0.001 | 0.98 | F_(1,73)_ = 13.66 | < 0.001*** |
| vCA3 | F_(1,69)_ = 35.47 | < 0.001*** | F_(1,69)_ = 60.72 | <0.001*** | F_(1,69)_ = 9.840 | 0.003 ** |
| Prl | F_(1,70)_ = 0.001 | 0.982 | H_1_ = 4.3 | 0.038* | F_(1,70)_ = 18.11 | < 0.001** |

*+p < 0.1, *p < 0.05, **p < 0.01, ***p < 0.001*

| Table S2. Main Effects of Condition on *Arc* Expression | | | | | | |
| --- | --- | --- | --- | --- | --- | --- |
| Cell Area | Nucleus | | Cytoplasm | | Dual | |
| Brain Region | Statistical value | p-value | Statistical value | p-value | Statistical value | p-value |
| lOFC | F_(2,57)_ = 0.57 | 0.57 | F_(2,57)_ = 0.64 | 0.534 | F_(2,57)_ = 0.07 | 0.932 |
| vOFC | F_(2,64)_ = 0.30 | 0.741 | F_(2,64)_ = 0.39 | 0.679 | F_(2,64)_ = 0.23 | 0.795 |
| ACC | F_(2,66)_ = 1.02 | 0.368 | F_(2,66)_ = 0.04 | 0.963 | F_(2,66)_ = 0.34 | 0.715 |
| Ifl | F_(2,73)_ = 0.21 | 0.815 | H_2_ = 0.23 | 0.89 | F_(2,73)_ = 1.42 | 0.248 |
| vCA3 | F_(2,69)_ = 1.35 | 0.265 | F_(2,69)_ = 0.01 | 0.989 | F_(2,69)_ = 0.27 | 0.764 |
| Prl | F_(2,70)_ = 0.005 | 0.995 | H_2_ = 2.19 | 0.335 | F_(2,70)_ = 0.18 | 0.837 |

| Table S3. Condition x Sex Interactions effects on *Arc* Expression | | | | | | |
| --- | --- | --- | --- | --- | --- | --- |
| Cell Area | Nucleus | | Cytoplasm | | Dual | |
| Brain Region | Statistical value | p-value | Statistical value | p-value | Statistical value | p-value |
| lOFC | F_(2,57)_ = 1.595 | 0.212 | F_(2,57)_ = 0.125 | 0.88 | F_(2,57)_ = 1.08 | 0.345 |
| vOFC | F_(2,64)_ = 2.452 | 0.094+ | F_(2,64)_ = 1.022 | 0.366 | F_(2,57)_ = 1.124 | 0.331 |
| ACC | F_(2,66)_ = 0.118 | 0.889 | F_(2,66)_ = 0.041 | 0.96 | F_(2,66)_ = 0.062 | 0.94 |
| Ifl | F_(2,73)_ = 0.365 | 0.696 | H_5_ = 3.25 | 0.662 | F_(2,73)_ = 0.763 | 0.47 |
| vCA3 | F_(2,69)_ = 0.996 | 0.375 | F_(2,69)_ = 0.66 | 0.519 | F_(2,69)_ = 0.011 | 0.99 |
| Prl | F_(2,70)_ = 3.963 | 0.023* | H_2_ = 8.45 | 0.133 | F_(2,70)_ = 0.32 | 0.727 |

*+p < 0.1, *p < 0.05*

| Table S4. Combined Food Task and Condition Effects on *Arc* Expression | | | | | | |
| --- | --- | --- | --- | --- | --- | --- |
| Cell Area | Nucleus | | Cytoplasm | | Dual | |
| Brain Region | Statistical value | p-value | Statistical value | p-value | Statistical value | p-value |
| lOFC | F_(4,58)_ = 0.567 | 0.688 | F_(4,58)_ = 0.818 | 0.519 | F_(4,58)_ = 0.233 | 0.918 |
| vOFC | F_(4,65)_ = 0.115 | 0.98 | F_(4,65)_ = 0.483 | 0.748 | F_(4,65)_ = 1.08 | 0.376 |
| ACC | F_(4,67)_ = 0.796 | 0.53 | F_(4,67)_ = 0.12 | 0.975 | F_(4,67)_ = 0.426 | 0.79 |
| Ifl | F_(4,74)_ = 0.116 | 0.977 | F_(4,74)_ = 0.24 | 0.915 | H_(4)_ = 4.85 | 0.304 |
| vCA3 | F_(4,70)_ = 1.51 | 0.209 | F_(4,70)_ = 0.653 | 0.627 | F_(4,70)_ = 0.729 | 0.575 |
| Prl | F_(4,70)_ = 0.377 | 0.824 | H_4_ = 2.27 | 0.686 | F_(4,70)_ = 1.120 | 0.354 |

| **Table S5. Group size by sex, condition, and brain region analyzed** | | | | | | |
| --- | --- | --- | --- | --- | --- | --- |
| **Brain**  **Region** | **Condition** | | | | | |
|  | Demonstrator | | Observer | | Control | |
|  | *Male* | *Female* | *Male* | *Female* | *Male* | *Female* |
| Prelimbic Cortex | n = 20 | n = 8 | n = 18 | n = 6 | n = 17 | n = 7 |
| Infralimbic Cortex | n = 19 | n = 9 | n = 19 | n = 8 | n = 15 | n = 9 |
| Ventral Orbitofrontal Cortex | n = 20 | n = 6 | n = 19 | n = 5 | n = 17 | n = 3 |
| Lateral Orbitofrontal Cortex | n = 20 | n = 4 | n = 16 | n = 4 | n = 17 | n = 2 |
| Anterior Cingulate Cortex (CG1) | n = 17 | n = 9 | n = 17 | n = 7 | n = 16 | n = 6 |
| vCA3 | n = 19 | n = 5 | n = 18 | n = 8 | n = 17 | n = 8 |
